# A socially assistive robot to support mental wellbeing in LGBTQ+ young people at risk of self-harm: a randomized controlled trial

**DOI:** 10.1038/s41591-026-04422-6

**Published:** 2026-06-04

**Authors:** A. Jess Williams, C. Aubrey Rhodes, Seonaid Cleare, Rohan Borschmann, James J. Gross, Kate Petrova, Lauren Posada, Christopher R. Tench, Amelia Chapman-Nisar, Lucy Martin, Chris Hollis, Ellen Townsend, Petr Slovak

**Affiliations:** 1 Department of Informatics, King’s College London, London, UK; 2 National Centre for Suicide Prevention and Self-Harm Research, Swansea University Medical School, Swansea, UK; 3 Institute of Mental Health, University of Nottingham, Nottingham, UK; 4 University of Glasgow, Glasgow, UK; 5 Health Service and Population Research Department, Institute of Psychiatry, Psychology & Neuroscience, King’s College London, London, UK; 6 Department of Psychiatry, University of Oxford, Oxford, UK; 7 Oxford Health NHS Foundation Trust, Oxfordshire, UK; 8 Justice Health Group, School of Population Health, Curtin University, Perth, Western Australia Australia; 9 Centre for Adolescent Health, Murdoch Children’s Research Institute, Royal Children’s Hospital, Melbourne, Victoria Australia; 10 Department of Psychology, Stanford University, Stanford, CA USA; 11 Division of Clinical Neuroscience, University of Nottingham, Nottingham, UK; 12 National Institute of Health Research (NIHR) MindTech MedTech Research Centre, Institute of Mental Health, School of Medicine, University of Nottingham, Nottingham, UK; 13 Nottinghamshire Healthcare NHS Foundation Trust, Nottingham, UK; 14 School of Psychology, University of Nottingham, Nottingham, UK

**Keywords:** Outcomes research, Psychology

## Abstract

LGBTQ+ youth commonly have unmet mental health needs and are at elevated risk for self-harm, yet many face persistent institutional barriers to accessing support. One impactful way to reduce risk and promote wellbeing is by supporting emotion regulation; that is, the process by which individuals can influence which emotions they feel, when they feel them and how they experience or express these emotions. This universal, modifiable process is widely considered a key transdiagnostic target for mental ill-health prevention and intervention efforts. We conducted a randomized controlled trial using Purrble, a socially assistive robot designed to provide in-the-moment emotion regulation support through intuitive tactile interaction. Between 12 January and 1 September 2024, 153 LGBTQ+ youth with self-harm ideation were randomized 1:1 to receive Purrble and safety planning (Purrble + SP) or safety planning alone (SP-Only), stratified by gender identity (50.3% transgender/gender diverse). Data were collected over 13 weeks, with data collection closing on 22 October 2024. The primary outcome was perceived emotion regulation difficulties at follow-up, adjusted for baseline, gender identity and age. Participants allocated to the Purrble intervention reported fewer emotion regulation difficulties at follow-up than those allocated to safety planning alone (adjusted mean difference: –3.04; 95% confidence interval (CI): −4.92 to −1.16; *P* = 0.002; partial *η*^2^ = 0.07). For secondary outcomes, participants in the Purrble intervention also reported significantly lower symptoms of anxiety and depression, but no significant main effect was observed for self-harm. No serious Purrble-related adverse events were observed. Purrble may offer a scalable intervention to complement existing therapeutic approaches to support LGBTQ+ youth to enhance their emotion regulation. ClinicalTrials.gov: NCT06025942.

## Abstract (web summary)

A randomized controlled trial integrating Purrble, a socially assistive tactile robot, into safety planning found that LGBTQ+ youth at risk of self-harm experienced improvements in Difficulties in Emotion Regulation Scale and fewer depressive symptoms compared to safety planning alone.

## Main

Lesbian, gay, bisexual, transgender, queer or questioning (LGBTQ+) youth are at increased risk for poor mental health outcomes, including elevated rates of depression and anxiety^1,2^, self-harm^3,4^ and suicidality^1,3,4^. In the United Kingdom (UK), an estimated 65.3% of LGBTQ+ youth have a history of self-harm^5^. Internationally, LGBTQ+ youth are also at increased risk of suicide^6^, being 3−6 times more likely to attempt suicide than their cisgender, heterosexual peers^4,7^.

Despite their heightened risk of poor mental health outcomes, LGBTQ+ youth are less likely than their non-LGBTQ+ peers to seek or receive treatment in traditional service settings^8^, due to both individual and systemic barriers^9–11^. In particular, LGBTQ+ youth who self-harm report experiencing additional barriers to treatment, exacerbated by fears relating to the disclosure of self-harm, including loss of personal agency, negative provider responses and being dismissed and/or stigmatized^8,10,12,13^. Such barriers highlight a critical need for innovative interventions for LGBTQ+ youth at risk of self-harm outside of traditional service contexts.

Digital mental health interventions are widely proposed as a scalable solution to address these gaps in support, and, among LGBTQ+ youth, such interventions are generally rated as feasible and acceptable^14^. A recent review suggests that digital approaches embedded in service settings (for example, Zoom-based therapy with a provider) and/or providing explicit psychoeducation and skill development (for example, self-guided adaptations of evidence-based therapies) show the most promising evidence for reducing psychopathology in LGBTQ+ youth, although none has evaluated self-harm as an outcome^15^. Digital supports that lack formal, structured components remain underdeveloped and, when evaluated in this population, tend to show weaker and highly variable effects^15^. Outside of the context of tightly controlled trials, engagement and retention with such digital interventions in real-world settings are often insufficient to derive clinical benefit^16^, reflecting systemic barriers to access, privacy and sustained use. This mismatch between the promise of these interventions and their low real-world uptake underscores the need for approaches that promote clinically meaningful skills and are intrinsically easy to weave into real-world routines.

**Fig. 1:**
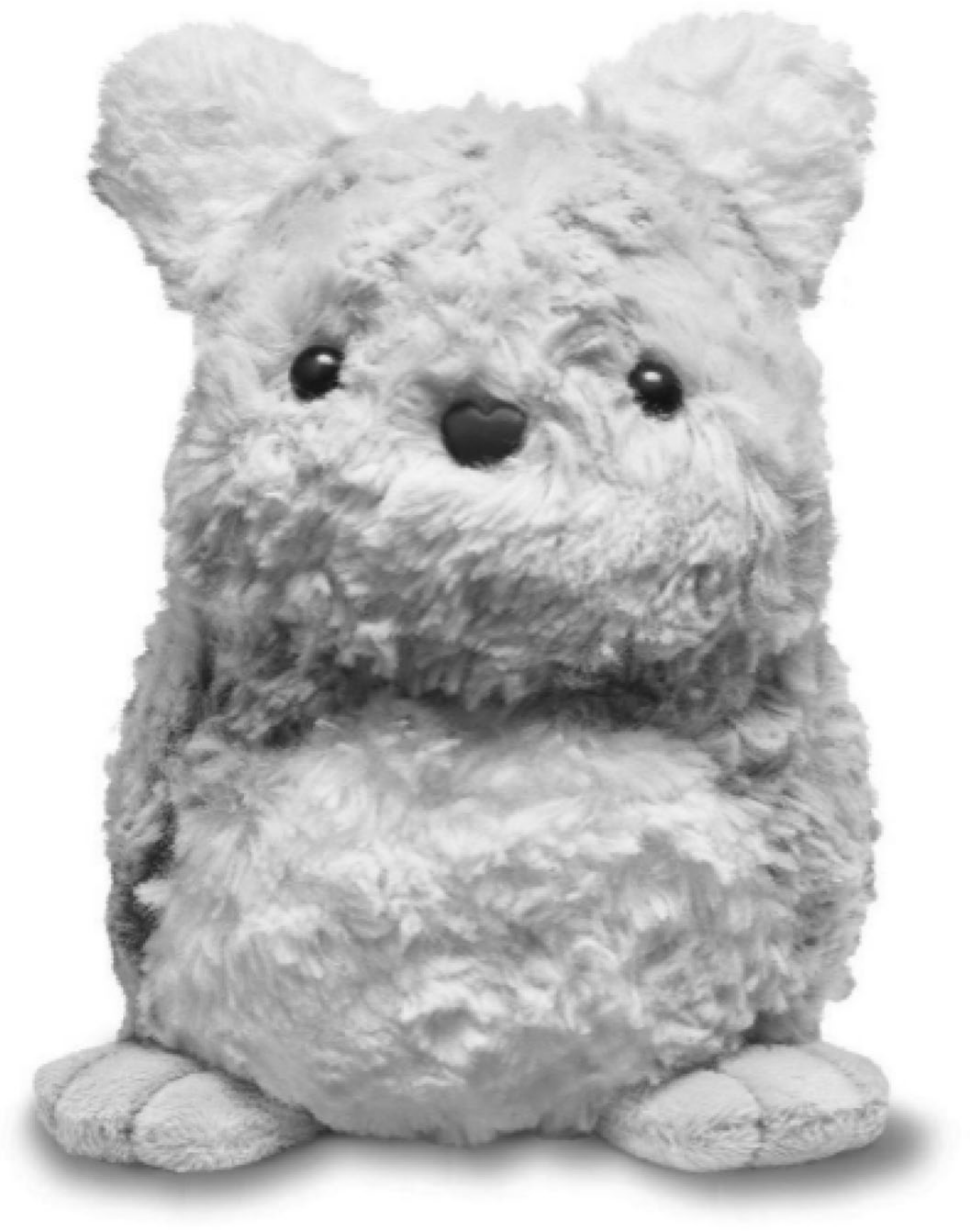
Purrble: socially assistive robot intervention device. Image of the socially assistive robot provided to participants in the intervention condition. Interactive features include touch-responsive sensors in the ears, feet and back stripe, a gyroscope for movement detection and an internal vibration motor that generates heartbeat-like and purr-like sensations.

One emerging and promising approach involves the use of socially assistive robots, which provide support to a user through social interactions. Socially assistive robots have been evaluated in various contexts, including education, family and healthcare settings with children^17–20^, and have shown encouraging outcomes in enhancing motivation, supporting skill development and improving predictors of poor mental health, such as loneliness and stress^17–22^. One notable example is Purrble (Fig. 1), a plush, socially assistive robot developed through co-design with youth^23^.

The primary target of Purrble is emotion regulation, which is the activation of a goal to influence emotion generation^24,25^. In practical terms, this involves influencing which emotions arise, when they occur and how these emotions are experienced or expressed^26^. All individuals engage in emotion regulation in response to everyday stressors, a process involving multiple stages, including identifying emotions, selecting and implementing strategies and monitoring their effects over time^27^. Variation in how this process unfolds, such as which strategies are used and in which context, has been shown to either support psychological wellbeing or increase risk of experiencing a range of mental health difficulties, in addition to self-harm^28–33^. As emotion regulation is a transdiagnostic process relevant across both clinical and non-clinical populations, it represents a powerful target for prevention and intervention^27,34^.

Unlike traditional emotion regulation interventions that rely on in-session, therapist-guided skill building and psychoeducation^35^, Purrble is designed to support emotion regulation in real-world settings through in-the-moment, intuitive, self-directed interaction, requiring no explicit instruction or prior orientation^17,23^. Specifically, Purrble targets early and late stages of the emotion regulation process, redirecting attention away from distressing stimuli and promoting physiological calming through tactile engagement, thereby supporting immediate downregulation of emotional arousal^27,31^. Purrble simulates a heartbeat that responds to touch, transitioning from frantic vibrations to a calming ‘purr’ as it is soothed by the user (see the intervention section in the Methods for a more thorough description). This real-time intervention offers private and consistently accessible support that may be especially valuable for LGBTQ+ youth who face barriers to accessing traditional mental health services. Indeed, LGBTQ+ youth at risk of self-harm have indicated that engaging with Purrble is both feasible and acceptable, with recent pilot work demonstrating its potential to interrupt self-harmful thoughts and behaviors by promoting in-the-moment implementation of emotion regulation strategies^36^. However, to date, no randomized controlled trials evaluating the efficacy of the Purrble intervention have been conducted.

In the current preregistered trial (ClinicalTrials.gov: NCT06025942), we evaluated the efficacy of access to Purrble and safety planning (Purrble + SP) in improving emotion regulation and reducing mental health symptom severity in a 13-week trial with LGBTQ+ youth experiencing thoughts of self-harm compared to safety planning alone (SP-Only). Safety planning, a brief collaborative intervention involving identifying warning signs, coping strategies and sources of support^37^, is recommended by multiple guiding bodies as standard (‘treatment as usual’) care for self-harm and suicidal presentations across practice settings^38,39^. For this reason, we included safety planning as a standard safeguarding procedure across conditions. The primary objective was to determine the intervention’s impact on emotion regulation (as measured by Difficulties in Emotion Regulation Scale-8 (DERS-8)). Secondary objectives evaluated intervention effects on key clinical outcomes: (1) self-harm (Self-Harm Questionnaire (SHQ)), (2) anxiety symptoms (Generalized Anxiety Disorder Scale-7 (GAD-7)) and (3) depression symptoms (Patient Health Questionnaire-9 (PHQ-9)). Post hoc analyses examined whether gender identity moderated intervention efficacy, thereby addressing a limitation of previous research, which has often grouped together heterogeneous groups of sexual orientation and gender identity minority groups, potentially obscuring unique experiences and intervention needs of transgender youth^40^.

## Results

### Participant disposition

Between 12 January and 1 September 2024, 556 individuals registered their interest in the trial, of whom 308 did not meet inclusion criteria or did not respond to study communications. In total, 248 youth completed the consent and demographics form, with 159 attending the compulsory safety planning session conducted by a doctoral-level member of the research team who had completed a 2-day Applied Suicide Intervention Skills Training (ASIST) suicide prevention workshop. The session was also used to verify participant information and allowed for the exclusion of two participants due to unverifiable participant information. After briefing, two more individuals were excluded; specifically, one withdrew and the other had an unverifiable postal address.

Participants were individually randomized in a parallel 1:1 ratio, using gender identity (cisgender versus transgender or gender diverse (TGD)) as a stratification factor to ensure balanced allocation across conditions at week 3. They were assigned to either the Purrble + SP condition or the SP-Only condition.

**Fig. 2:**
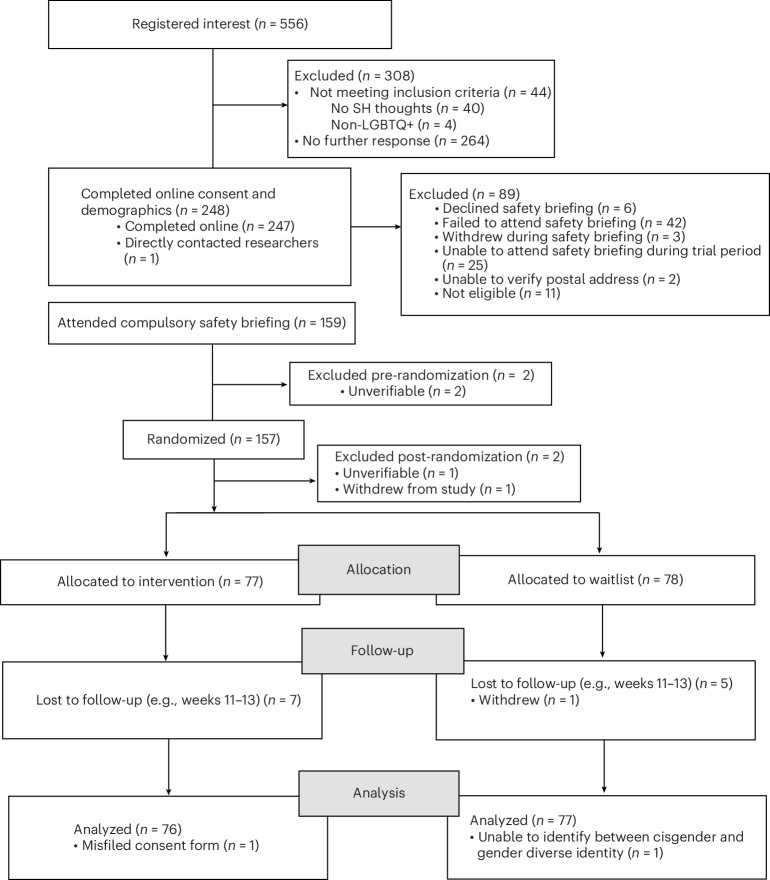
CONSORT flow diagram of participant progression through the trial. Diagram showing the numbers of participants assessed for eligibility, excluded, randomized to each study condition, retained at follow-up and included in the final analyses. SH, self-harm.

An additional two participants were excluded at the analysis stage due to misfiling errors within study records, resulting in a final analytic sample of 153 participants (Purrble + SP: *n* = 76; SP-Only: *n* = 77). Recruitment, randomization and follow-up numbers are summarized in the CONSORT diagram (Fig. 2). Final data collection occurred on 22 October 2024.

Demographic characteristics were similar across conditions. Participants in the Purrble + SP and SP-Only groups were of equivalent age at baseline (20.4 ± 2.3 years versus 20.1 ± 2.5years; *t*_150.5_ = 0.86, *P* = 0.39, Cohenʼs *d* = 0.14, 95% CI: −0.18 to 0.46). Both groups were predominantly white (78.9% versus 71.4%) and sexually diverse (Table 1). Gender identity was evenly distributed across the conditions, with 76 participants (49.7%) identifying as cisgender and 77 participants (50.3%) identifying as TGD. These characteristics are consistent with those typically reported in research involving LGBTQ+ youth who engage in self-harm^1,5,36^. The elevated representation of gender-diverse participants in this sample reflects the study’s recruitment priorities and design.

**Table 1:** Participant characteristics by condition

	Purrble + SP (*n* = 76)	SP-Only (*n* = 77)
Age (years), mean (s.d.)	20.4 (2.3)	20.1 (2.5)
Gender identity, *n* (%)		
Cisgender	39 (51.3)	37 (48.1)
TGD	37 (48.7)	40 (51.9)
Sexual orientation, *n* (%)		
Asexual	13 (17.1)	9 (11.7)
Bisexual	28 (36.8)	25 (32.5)
Demisexual	2 (2.6)	1 (1.3)
Gay/lesbian	11 (14.5)	18 (23.4)
Heterosexual	1 (1.3)	0 (0)
Pansexual	8 (10.5)	9 (11.7)
Queer	13 (17.1)	15 (19.5)
Race/ethnicity, *n* (%)		
Arabic	0 (0)	1 (1.3)
Asian	10 (13.2)	17 (22.1)
Black	1 (1.3)	3 (3.9)
Hispanic	2 (2.6)	0 (0)
White	60 (78.9)	55 (71.4)
Unknown	9 (11.8)	5 (6.5)

Five participants in the Purrble + SP condition and four participants in the SP-Only condition reported multiple racial identities. Participants were coded as TGD if they reported being transgender, trans-masculine/feminine, non-binary, agender, gender queer, questioning their gender, genderfluid, demiboy/girl or any combination of these identities. Sexual orientation and race/ethnicity were self-reported using open-text responses and subsequently coded into discrete categories.

Participants completed an average of 12.4 questionnaires in the Purrble + SP condition and 12.9 questionnaires in the SP-Only condition out of a possible 14 (intake (‘week 0’) through follow-up (‘week 13’)). Attrition was low overall, with 9.2% attrition in the Purrble + SP condition (seven out of 76 participants) and 6.5% attrition in the SP-Only condition (five out of 77 participants). Across the full sample, regression analyses indicated a significant decline in weekly survey response over time, with the average number of weekly respondents decreasing by approximately 2.16 per week (s.e. = 0.29, *t* = −7.39, *P* < 0.001). When examined by condition, survey response declined at a rate of −1.46 per week in the Purrble + SP group (s.e. = 0.23, *t* = −6.22, *P* < 0.001) and at a rate of −0.70 per week in the SP-Only group (s.e. = 0.12, *t* = −5.82, *P* < 0.001) (see Extended Data Table 1 for rates over time). A time × condition interaction (unstandardized regression coefficient (B) = 0.76, s.e. = 0.26, *P* = 0.009, 95% CI: 0.21−1.30) suggested a significantly steeper rate of response decline in the Purrble + SP group. We accounted for this difference in the sensitivity analyses.

### Primary outcome: emotion regulation

Baseline (weeks 1−3) and follow-up (weeks 11−13) descriptive statistics are presented in Table 2. We fit an analysis of covariance (ANCOVA) predicting follow-up emotion regulation difficulties from condition, gender identity, baseline emotion regulation and age (Table 3). There was a significant main effect of condition such that participants assigned to Purrble + SP reported fewer emotion regulation difficulties as measured by DERS-8 at follow-up (weeks 11−13) than those assigned to SP-Only (B = −3.04, 95% CI: −4.92 to −1.16, s.e. = 0.95, *P* = 0.002; partial *η*^2^ = 0.07), controlling for baseline, gender identity and age. In this main effects model, gender identity and age were not significant predictors of follow-up DERS-8 scores. Analysis of clinically reliable change (Table 2) favored Purrble + SP, with significantly higher odds of reliable improvement in DERS-8 scores compared to SP-Only (29% versus 14%; odds ratio = 2.44, 95% CI: 1.09−5.49, *P* = 0.03). Rates of reliable decline did not differ significantly between groups.

**Table 2:** Descriptive statistics and rates of clinically reliable change for primary and secondary outcomes

Outcome	Condition	*N*	Mean (s.d.)		Reliable improvement			Reliable decline		
			Baseline	Follow-up	*n* (%)	OR [95% CI]	*P*	*n* (%)	OR [95% CI]	*P*
Emotion regulation	Purrble + SP	76	27.92 (5.10)	25.26 (7.80)	22 (28.9%)	2.44 [1.09−5.49]	0.03	7 (9.2%)	0.68 [0.24−1.89]	0.46
	SP-Only	77	28.38 (4.32)	28.61 (6.52)	11 (14.3%)			10 (13.0%)		
Depression	Purrble + SP	76	15.39 (4.90)	13.44 (6.66)	24 (31.6%)	4.62 [1.85−11.53]	0.001	7 (9.2%)	0.68 [0.24−1.89]	0.46
	SP-Only	77	14.70 (4.24)	15.15 (5.93)	7 (9.1%)			10 (13.0%)		
Anxiety	Purrble + SP	76	13.78 (4.25)	12.00 (5.47)	22 (28.9%)	2.01 [0.92−4.36]	0.079	8 (10.5%)	0.71 [0.27−1.87]	0.48
	SP-Only	77	13.65 (3.74)	13.20 (4.46)	13 (16.9%)			11 (14.3%)		

Data are presented as mean (s.d.) or frequency (%). *n* indicates the number of participants. Odds ratios (ORs) and *P* values represent the likelihood of reliable improvement or decline in the Purrble condition relative to the SP-Only condition.

**Table 3:** Purrble effects on emotion regulation, depression and anxiety

Predictor	B	95% CI		s.e.	*t*	*P*	Partial *η*^2^	95% CI	
		Lower limit	Upper limit					Lower limit	Upper limit
Emotion regulation									
(Intercept)	−0.96	−11.07	9.15	5.11	−0.19	0.852	—	—	—
Condition	−3.04**	−4.92	−1.16	0.95	−3.20	0.002	0.07	0.01	0.17
Baseline emotion regulation	0.92***	0.72	1.12	0.10	9.21	<0.001	0.39	0.26	0.49
Gender identity	1.69	−0.26	3.64	0.99	1.72	0.088	0.02	0.00	0.09
Age	0.13	−0.29	0.54	0.21	0.60	0.549	0.00	0.00	0.05
Depression									
(Intercept)	−5.62	−12.58	1.34	3.52	−1.60	0.113	—	—	—
Condition	−2.60***	−4.02	−1.19	0.71	−3.64	<0.001	0.09	0.02	0.19
Baseline depression	1.00***	0.85	1.16	0.08	12.96	<0.001	0.55	0.45	0.64
Gender identity	0.25	−1.22	1.73	0.75	0.34	0.734	0.00	0.00	0.04
Age	0.29	−0.02	0.61	0.16	1.86	0.064	0.03	0.00	0.10
Anxiety									
(Intercept)	−2.72	−9.15	3.71	3.25	−0.84	0.404	—	—	—
Condition	−1.35*	−2.66	−0.04	0.66	−2.04	0.044	0.03	0.00	0.11
Baseline anxiety	0.74***	0.58	0.90	0.08	8.98	<0.001	0.37	0.25	0.48
Gender identity	0.75	−0.62	2.12	0.69	1.08	0.281	0.01	0.00	0.06
Age	0.27	−0.02	0.56	0.15	1.84	0.068	0.02	0.00	0.10

Results are from two-sided ANCOVA models estimated using linear regression, with post-intervention outcome regressed on condition, baseline outcome, gender identity and age. Gender identity was coded as 0 = cisgender, 1 = TGD. Condition was coded as 0 = SP-Only, 1 = Purrble + SP. Partial *η*^2^ reflects the effect size for each predictor. *P < 0.05, **P < 0.01, ***P < 0.001. The test statistic reported for each coefficient is *t* with 134 residual degrees of freedom for all models. To evaluate robustness to multiple testing, Benjamini–Hochberg correction was applied across the three primary and secondary continuous outcome models; significance was unchanged after correction (adjusted *P* values = 0.003, <0.001 and 0.044).

### Secondary outcomes

#### Self-harm

No significant main effects on self-harm were observed as measured by SHQ screening questions (Extended Data Table 2).

#### Depression

Descriptive statistics for depressive symptoms, as measured by the PHQ-9, are presented in Table 2. We fit an ANCOVA predicting follow-up depressive symptom severity from condition, gender identity, baseline depressive symptoms and age (Table 3). There was a significant main effect of condition such that participants assigned to Purrble + SP reported lower depressive symptom severity at follow-up than those assigned to SP-Only (B = −2.60, 95% CI: −4.02 to −1.19, s.e. = 0.71, *P* < 0.001; partial *η*^2^ = 0.09), controlling for baseline, gender identity and age. In this main effects model, neither gender identity nor age were significant predictors of follow-up scores. Examinations of clinically reliable change favored Purrble + SP, indicating significantly higher odds of reliable improvement in the Purrble + SP condition (odds ratio = 4.62, 95% CI: 1.85−11.53, *P* = 0.001) with no difference in reliable decline (Table 2).

#### Anxiety

Descriptive statistics for anxiety symptoms as measured by the GAD-7 questionnaire are presented in Table 2. We fit an ANCOVA predicting follow-up anxiety symptom severity from condition, gender identity, baseline anxiety and age (Table 3). There was a significant main effect of condition, indicating lower follow-up anxiety symptom severity for participants in the Purrble + SP condition compared to SP-Only (B = −1.35, 95% CI: −2.66 to −0.04, s.e. = 0.66, *P* = 0.044; partial *η*^2^ = 0.03), controlling for baseline, gender identity and age. Gender identity and age did not significantly predict the severity of anxiety symptoms at follow-up. However, analyses of clinically reliable change indicated that odds of improvement (odds ratio = 2.01, 95% CI: 0.92−4.36, *P* = 0.079) did not differ significantly between conditions (Table 2).

### Safety

Three participants required reactive safeguarding for adverse events during the trial: one from the Purrble + SP condition and two from the SP-Only condition. During safeguarding contacts, participants attributed their increased distress to life events external to the trial, and no imminent risk of self-harm or suicide was identified. However, participants in the Purrble + SP condition reported feeling guilty about not engaging with the device. They were reassured that there is no ‘right’ way to use Purrble, encouraged to switch off the device and advised to reengage only if they felt comfortable. No serious adverse events occurred.

### Exploratory analyses

#### Emotion regulation

Following the preregistered plan, we fit a linear mixed effects model with random intercepts and slopes for week, including fixed effects of week, condition and their interaction and controlling for gender identity and age (Extended Data Table 3). Emotion regulation difficulties declined over time on average (B = −0.12, *P* = 0.007), and the week × condition interaction was significant (B = −0.14, *P* = 0.002), indicating different rates of change over time by condition. Probing the interaction indicated that emotion regulation difficulties declined in the Purrble + SP condition by approximately 0.27 points per week, whereas change in the SP-Only condition was minimal.

#### Depression

Following the preregistered plan, we fit a linear mixed effects model with random intercepts and slopes for week, including fixed effects of week, condition and their interaction and controlling for gender identity and age (Extended Data Table 3). Depressive symptoms declined over time on average (B = −0.07, *P* = 0.045), and the week × condition interaction was significant (B = −0.11, *P* = 0.001), indicating different rates of change over time by condition. Probing the interaction indicated that depressive symptoms declined in the Purrble + SP condition by approximately 0.18 points per week, whereas change in the SP-Only condition was minimal. Unlike the ANCOVA models, this model also showed a significant main effect of gender identity (B = −0.82, *P* = 0.034), indicating overall differences in depressive symptoms by gender identity.

#### Anxiety

Following the preregistered exploratory analysis, we fit a linear mixed effects model with random intercepts and slopes for week, including fixed effects of week, condition and their interaction and controlling for gender identity and age (Extended Data Table 3). Anxiety symptoms declined over time on average (B = −0.11, *P* = 0.001). However, the week × condition interaction was not significant (B = −0.05, *P* = 0.119), indicating that rates of change over time did not differ significantly by condition. The main effect of gender identity was not significant (B = −0.63, *P* = 0.052).

### Interaction analyses

Condition by gender identity interaction analyses were added after publication of the protocol but were prespecified prior to conducting any analyses. As such, interactive analyses are considered post hoc.

#### Emotion regulation

To test whether the Purrble + SP effect was moderated by gender identity, we fit a second ANCOVA that included a condition × gender identity interaction term (Table 4). This model revealed a significant interaction (B = 3.92, 95% CI: 0.22−7.63, s.e. = 1.87, *P* = 0.038, partial *η*^2^ = 0.03). There was no main effect of gender identity. We probed the interaction using simple slopes analyses, which revealed a significant benefit for cisgender participants in the Purrble + SP condition compared to the SP-Only condition (B = −5.01, s.e. = 1.33, *P* < 0.001). By contrast, the conditional treatment effect for TGD participants was not significant (B = −1.08, s.e. = 1.32, *t* = −0.82, *P* =0.42). Estimated marginal means supported this pattern (cisgender: Purrble + SP mean = 23.6 versus SP-Only mean = 28.6; TGD: Purrble + SP mean = 27.3 versus SP-Only mean = 28.4; Extended Data Table 4).

**Table 4:** Gender identity by condition interactive effects on emotion regulation, depression and anxiety

Predictor	B	95% CI		s.e.	*t*	*P*	Partial *η*^2^	95% CI	
		Lower limit	Upper limit					Lower limit	Upper limit
Emotion regulation									
(Intercept)	0.32	-9.75	10.38	5.09	0.06	0.951	—	—	—
Condition	−5.01***	−7.63	−2.38	1.33	−3.77	<0.001	0.10	0.02	0.20
Baseline emotion regulation	0.91***	0.72	1.11	0.10	9.24	<0.001	0.39	0.27	0.50
Gender identity	−0.24	−2.90	2.41	1.34	−0.18	0.857	0.00	0.00	0.03
Age	0.12	−0.29	0.53	0.21	0.59	0.558	0.00	0.00	0.05
Condition × gender identity	3.92*	0.22	7.63	1.87	2.10	0.038	0.03	0.00	0.11
Depression									
(Intercept)	−4.72	−11.70	2.26	3.53	−1.34	0.183	—	—	—
Condition	−3.86***	−5.84	−1.89	1.00	−3.87	<0.001	0.10	0.02	0.20
Baseline depression	0.99***	0.83	1.14	0.08	12.79	<0.001	0.55	0.44	0.63
Gender identity	−0.98	−2.99	1.02	1.01	−0.97	0.334	0.01	0.00	0.06
Age	0.29	-0.02	0.60	0.16	1.86	0.065	0.03	0.00	0.10
Condition × gender identity	2.54	−0.27	5.35	1.42	1.79	0.076	0.02	0.00	0.10
Anxiety									
(Intercept)	−1.82	−8.21	4.58	3.23	−0.56	0.575	—	—	—
Condition	−2.78**	−4.61	−0.95	0.92	−3.00	0.003	0.06	0.01	0.16
Baseline anxiety	0.73***	0.57	0.89	0.08	8.95	<0.001	0.37	0.25	0.48
Gender identity	−0.65	−2.51	1.20	0.94	−0.69	0.489	0.00	0.00	0.05
Age	0.27	−0.02	0.56	0.15	1.85	0.067	0.02	0.00	0.10
Condition × gender identity	2.86*	0.27	5.45	1.31	2.18	0.031	0.03	0.00	0.11

Results are from two-sided ANCOVA models estimated using linear regression, with post-intervention outcome regressed on condition, baseline outcome, gender identity, age and the condition × gender identity interaction. Gender identity was coded as 0 = cisgender, 1 = TGD. Condition was coded as 0 = SP-Only, 1 = Purrble + SP. Partial *η*^2^ reflects effect size for each predictor. *P < 0.05, **P < 0.01, ***P < 0.001. The test statistic reported for each coefficient is *t*; all coefficient tests used 134 residual degrees of freedom. Benjamini–Hochberg correction was applied across the three exploratory moderation models examining the condition × gender identity interaction; none of the interaction effects remained statistically significant after correction (adjusted *P* values = 0.057, 0.076 and 0.057).

#### Self-harm

No significant condition × gender identity interaction effects were observed on self-harm.

#### Depression

To test whether the effect on depressive symptom severity was moderated by gender identity, we fit a post hoc ANCOVA that included a condition × gender identity interaction term (Table 4). The interaction was not significant (B = 2.54, s.e. = 1.42, *P* = 0.076, partial *η*^2^ = 0.023).

#### Anxiety

To test moderation by gender identity, we ran a post hoc model in which we added a condition × gender identity term (Table 4). The interaction was significant (B = 2.86, 95% CI: 0.27−5.45, s.e. = 1.31, *P* = 0.031, partial *η*^2^ = 0.03), whereas the main effect of gender identity remained non-significant. Simple slopes analyses showed a significant Purrble + SP benefit for cisgender participants (B = −2.78, s.e. = 0.92, *P* = 0.003) but not for TGD participants (B = 0.08, s.e. = 0.93, *P* = 0.93). Estimated marginal means mirrored this pattern (cisgender: Purrble + SP mean ≈ 10.8 versus SP-Only mean ≈ 13.6; TGD: Purrble + SP mean ≈ 13.0 versus SP-Only mean ≈ 12.9; Extended Data Table 4).

### Mechanistic outcome analyses

For the preregistered main effects analyses of hypothesized mechanistic outcomes (hope, loneliness and attentional deployment), we fit ANCOVA models predicting each follow-up outcome from condition, baseline outcome, gender identity and age. No significant main effects were observed (Extended Data Table 5). We then conducted post hoc moderation analyses parallel to those used for the primary and secondary outcomes, adding a condition × gender identity interaction term to each model (Extended Data Table 6). One significant interaction was identified: the effect of condition on hope agency differed by gender identity, with improvement evident among cisgender participants but not TGD participants in the Purrble + SP condition.

### Sensitivity analyses

Baseline measures of outcome variables and participant age did not differ significantly between conditions. We examined potential multivariate outliers among baseline variables (emotion regulation, depression and anxiety) using Mahalanobis distance (D^2^).Distances were compared to the *χ*^2^ distribution with 3 degrees of freedom (critical value = 11.34). One participant exceeded this threshold (*D*^2^ = 14.57). To evaluate influence on model results, we reran all primary and secondary analyses (ANCOVA and linear mixed effects models) with and without this participant. The pattern, magnitude and significance of results were unchanged. Accordingly, all analyses were reported using the full sample.

To evaluate robustness to multiple testing, we applied the Benjamini−Hochberg correction separately within each family of analyses. Specifically, we corrected for the three primary and secondary continuous outcomes (DERS-8, GAD-7 and PHQ-9) and, in a separate step, for the three moderation models examining condition × gender identity interactions. The moderation analyses were exploratory and not preregistered. For the continuous outcomes, significance levels were unchanged after correction (adjusted *P*values = 0.003, <0.001 and 0.044). For the moderation analyses, none of the effects remained statistically significant after correction (adjusted *P*values = 0.057, 0.076 and 0.057).

To assess the robustness to differential survey completion over time, we conducted several sensitivity analyses examining whether attrition or survey completion was associated with condition, gender identity or baseline symptom levels. Binary attrition rates did not differ significantly by condition, *χ*^2^_1_ = 0.11, *P* = 0.75, or by gender identity, *χ*^2^_1_ < 0.01, *P* > 0.99. In addition, attrition status was not significantly associated with baseline variables nor did these associations vary by condition. As an additional sensitivity analysis, we reestimated all the main effects (ANCOVA) models including total number of survey responses as a covariate; this did not materially change the significance, magnitude or direction of the primary or secondary findings.

### Post hoc analysis

To elucidate potential reasons for the differential efficacy observed between cisgender and TGD youth, we examined whether perceptions of Purrble engagement/fit differed by gender identity over time. There was no baseline difference in perceived engagement/fit of Purrble by gender identity (B = −0.06, s.e. = 0.10, *P* = 0.54). However, a significant week × gender identity interaction (B = 0.017, s.e. = 0.006, *t*_531_ = 2.93, *P* = 0.004) indicated that Purrble engagement/fit declined significantly faster for TGD youth. Specifically, TGD youth showed a steeper decline in perceived engagement/fit (simple slope B = −0.056, 95% CI: −0.073 to −0.040) compared to their cisgender peers (*N* = −0.022, 95% CI: −0.038 to −0.005).

## Discussion

This randomized controlled trial examined the effects of safety planning plus Purrble, an in situ, socially assistive robot, for enhancing emotion regulation compared to safety planning alone among LGBTQ+ youth with thoughts of self-harm. All participants engaged in a structured safety planning session at baseline, mirroring many real-world care pathways in which youth are offered a single episode of safety planning with little or no structured follow-up^38,41^. At follow-up, participants in the intervention arm showed reductions in emotion regulation difficulties, anxiety and depressive symptoms but not significant changes in self-harm thoughts or behaviors. The emotion regulation effect size was medium to large, mirroring meta-analytic effects for skills-based interventions^42^ and aligning with our a priori benchmark.

Crucially, Purrble delivered these benefits without therapist contact, explicit skills training or time-intensive engagement. In light of persistent barriers to care for LGBTQ+ youth^8–11^, an in situ, scalable intervention with transdiagnostic effects may be useful. From an economic perspective, the wholesale cost of providing Purrble (approximately £25 plus approximately £6 shipping per unit in the case of this trial) is a small fraction of the mean general hospital cost of a single self-harm episode in England (approximately £809)^43^. Taken together, Purrble may offer a distinct, scalable and low-burden intervention that is readily accessible and could help expand reach and advance equity.

We hypothesize that Purrble’s effects are driven by its unique interaction model—specifically, in situ, intuitive interactions that provide immediate regulatory support during naturally occurring emotionally salient moments, repeated over time. Consistent with the original logic model underpinning the intervention, Purrble is designed to target both early and late stages of the emotion regulation process (that is, attentional deployment and response modulation) by redirecting attention away from distressing stimuli and facilitating physiological calming through tactile interaction^17,27,31^. According to the proposed theory of change, repeated use of Purrble in emotionally salient moments may, over time, shape how individuals respond to emotional challenges, supporting the development of more adaptive regulation patterns. Specifically, individuals may become more inclined to redirect attention during heightened arousal, respond with self-soothing and develop a greater sense of agency in the management of their emotional experiences, each of which plays a central role in internalizing psychopathology^44^. Future work should directly evaluate these pathways, including whether frequency of use of in situ regulatory support, particularly matched against frequency of need, leads to durable changes in emotion regulation skills and beliefs over time and whether such changes account for subsequent improvements in anxiety and depression.

Post hoc interaction analyses indicated that gender identity moderated Purrble + SP effects on emotion regulation difficulties and anxiety. Specifically, we found that cisgender youth in the intervention condition showed significant improvements relative to those in the SP-Only condition, whereas effects were not evident for TGD youth. No moderation was observed for depression. Although these interaction effects were not robust to multiplicity adjustment (for example, Benjamini–Hochberg), the direction and consistency of the simple slope estimates suggest that subgroup differences by gender identity may be meaningful and warrant replication in larger, adequately powered trials. These patterns underscore heterogeneity within LGBTQ+ populations and the likelihood of differential needs across identity groups^40,43^. However, few intervention trials explicitly examine cisgender versus TGD differences^15^, often because of lower TGD participation^45,46^, limiting precision in estimating subgroup effects.

An important avenue for future work is to examine how TGD youth perceive and engage with a socially assistive robot like Purrble over time, in order to explain the subgroup patterns that we observed. Prior research indicates that TGD youth have distinct needs around identity, embodiment and social context^47,48^ and that these needs are often overlooked in designs that assume everyone’s gender identity aligns with their sex assigned at birth^49^. In post hoc analyses, TGD and cisgender participants did not differ at baseline in perceived Purrble fit. However, over the intervention period, TGD youth showed a steeper decline in perceived fit than cisgender youth. Future qualitative and mixed methods work could clarify how TGD youth interpret and use Purrble in daily life and which design features may need adaptation. A second research avenue is to consider Purrble as an adjunctive intervention. Beyond the present trial, which evaluated Purrble as an adjunct to a single-session safety plan and was intentionally designed to allow participants to seek any additional formal or informal support they desired, future work should examine how it can be embedded at multiple points along youth care pathways. Young people on waiting lists are at heightened risk of suicide attempt^50^ and often drop out or disengage before reaching treatment^51^, but offering waitlist interventions may help reduce such outcomes^52^. Purrble may support the development of emotion regulation strategies to scaffold engagement with therapy and reduce the impact of poor mental health outcomes. Furthermore, as Purrble is compatible with multiple theoretical approaches, it could work alongside a variety of evidence-based therapies, such as dialectical behavior therapy for adolescents (DBT-A)^53^.

Results should be interpreted in the context of several limitations. First, our design compared Purrble plus safety planning with safety planning alone, with the SP-Only group offered Purrble after follow-up. Although this approach was selected to ensure equitable access and to reflect advisory group preferences for mimicking the lived experience of receiving a single safety planning contact with little follow-up, it introduces methodological challenges. Second, representation of lived experience within the advisory group was limited, with only one member identifying as LGBTQ+ and no trans lived experience represented. Future research would benefit from deeper and more diverse LGBTQ+ involvement, particularly inclusion of trans individuals, in advisory roles and throughout the research process. Third, as the study was advertised using images of Purrble, there is a possibility of self-selection bias, with participants potentially more inclined toward engaging with comforting or ‘fluffy’ robotic companions. This may have influenced results, as participants with a preexisting interest in such technologies could be more receptive to the intervention or inclined toward its perceived impacts. Fourth, participants were aware of their group assignment and that the SP-Only group would later receive Purrble, creating potential expectancy and engagement differences that may inflate self-reported improvements and limit causal inference^54^. Fifth, there was no sham or placebo device to isolate Purrble-specific mechanisms (for example, socially assistive feedback) from non-specific factors such as novelty or having any comforting object. Finally, the lack of a long-term follow-up limits conclusions about the durability of effects.

Future trials would benefit from more active control conditions, such as clearly documented treatment-as-usual comparators^55^ or, where feasible, attention-matched or placebo device controls that more stringently test the added value of the socially assistive components of Purrble. A key challenge for developing an appropriate control lies in disentangling which specific functionalities (for example, tactile feedback, responsive social cues, physical comfort and routine use) most contribute to these in-the-moment regulation effects and whether these mechanisms operate similarly across users. Because these components are interwoven in the device’s interaction model, creating a credible placebo or matched alternative that preserves the non-specific elements while removing the active mechanisms remains methodologically complex. Furthermore, a larger, sufficiently powered trial for self-harm as a primary outcome is required to establish whether deployment of Purrble could be effective in preventing the escalation of emotional distress into self-harm thoughts and behaviors, a potential effect derived from our pilot work^36^.

This preregistered, stratified, randomized controlled trial provides evidence that an in situ socially assistive robot can improve mental health-relevant outcomes for LGBTQ+ youth with self-harm thoughts. Across 13 weeks, Purrble plus safety planning produced meaningful reductions in emotion regulation difficulties compared to safety planning alone, achieved without any therapist contact or explicit skills training. Taken together, these findings position Purrble as a possible adjunct to usual care or waitlist periods for cisgender sexual minority youth while underscoring the need for targeted co-design and evaluation to meet the distinct needs of TGD youth. To enhance generalizability and support real-world implementation, future work should employ placebo control conditions, test mechanisms of change, extend follow-up and evaluate integration alongside evidence-based therapies and service pathways.

## Methods

### Trial design and ethical approval

This was a parallel, stratified, randomized controlled trial conducted with LGBTQ+ youth in the UK. All study procedures were approved by the ethics committee at King’s College London (RESCM-22/23-34570), and all participants provided written informed consent prior to participation. All trial procedures, including anticipated safeguarding concerns related to psychological distress or potential adverse events associated with participation, were prospectively registered before enrollment of the first participant (ClinicalTrials.gov: NCT06025942, available at https://bmjopen.bmj.com/content/bmjopen/14/1/e079801.full.pdf, published 8 December 2023). As we anticipated potential adverse events associated with participation, explicit plans for identifying, monitoring and addressing these concerns were included (‘Safeguarding’ section). This paper was developed in accordance with the CONSORT 2010 checklist^56^, and we ensured transparency by prospectively publishing the detailed trial design and the protocol^57^.

### Participant recruitment

Participant recruitment occurred between January and September 2024, using diverse strategies including online platforms (Twitter/X, Instagram, paid research participation sites, university participant pools and MQ Mental Health Research (https://participate.mqmentalhealth.org/)), physical community outreach (schools and community centers) and snowball sampling methods (word of mouth and Volunteer Tutors Organization). This multichannel approach facilitated broad outreach among eligible youth. Recruitment was discontinued in September 2024 with final follow-ups conducted in October 2024 due to a substantial reduction in registration rates, suggesting saturation of available recruitment channels. The target minimum sample size had already been successfully achieved by this point.

Eligible participants were individuals aged 16–25 years (inclusive). The lower bound was chosen to begin at the UK age of consent and the upper bound to align with the National Youth Agency’s definition of youth (11–25 years)^58^. This range captures the developmental period when self-harm is likely to reach its peak^59,60^. Additional inclusion criteria included residing in the UK throughout the study duration, identifying within the sexual orientation or gender identity minority spectrum (LGBTQ+) and reporting any experiences of self-harm ideation or behavior within the previous month (that is, any response greater than ‘none’ on any questions on the screener), with no upper severity limit. The 1-month timeframe for self-harm ideation was selected to capture current and recent ideation, acknowledging the typically fluctuating nature of these experiences^61^. Additional inclusion criteria were proficiency in reading, writing and speaking English, which was essential for active engagement and accurate reporting within the study protocol. Individuals were excluded if they identified as cisgender and heterosexual, if they were above or below the age range (16-25 years inclusive), if they did not have recent experiences of self-harm ideation or behavior (in the last month) or if they lived outside of the UK.

It was not necessary for participants to indicate whether they were receiving any additional treatments or therapies for their mental health difficulties. This design decision was intentional to reflect real-world conditions in which individuals might use Purrble either alongside or independently of other supports or interventions.

### Procedures

#### Randomization

Randomization was conducted after consent and baseline data collection (week 3) by the study coordinator, who was the only person with access to the randomization process. Participants were grouped into blocks based on similar enrollment timeframes. Within each block, participants were individually randomized in a 1:1 ratio (stratified by gender identity and cisgender versus TGD, to ensure balance between conditions) to either the Purrble + SP condition or the SP-Only (plus waitlist) condition, using an online randomization generator. Participants were coded as TGD if they reported that they were transgender, trans-masculine/feminine, non-binary, agender, gender queer, questioning their gender, genderfluid, demiboy/girl or any combination of the above. Participants were notified of their condition allocation via email after the closure of the week 3 data collection window. On the same day, Purrble devices were dispatched to participants in the Purrble + SP condition via express delivery to ensure arrival prior to the start of the week 4 survey.

#### Trial procedures

Participants were randomized to their condition group and enrolled into the trial after completing a synchronous compulsory safety briefing and planning session via Zoom. These intake calls were conducted in private office spaces by the ASIST-trained researcher, and participants were asked to take the call in a private, quiet location of their choice. The trial period lasted 13 weeks, with weekly online surveys hosted by Qualtrics. Surveys were sent to participants via email, with a next-day automatic reminder from Qualtrics and a personalized message the following day to those who had not completed their survey. Surveys were open to be completed for 3 days.

The initial 3 weeks of the trial acted as baseline assessments, followed by randomization, with intervention participants receiving Purrble in the fourth week. The deployment period lasted for 10 weeks. An extended assessment was conducted at week 3 (final baseline timepoint), week 8 (fifth week of deployment) and week 13 (final week of the trial). Those in SP-Only received Purrble after week 13. All participants were able to keep their Purrbles after the trial was completed and were paid £5 for each completed survey.

#### Public and patient involvement

This project is supported by members of Sprouting Minds (https://digitalyouth.ac.uk/the-digital-youth-programme/about-sprouting-minds/), an advisory group comprising young individuals with lived experience of poor mental health, specifically involved in Digital Youth research initiatives. A subgroup of advisors from the wider Sprouting Minds network chose to engage more closely with this project due to shared identity characteristics and personal relevance to the study population, with approximately one-third of this subgroup self-identifying as LGBTQ+. Building on their foundational involvement in prior related research^36^, their input substantially shaped key design decisions throughout this trial design.

Advisors from Sprouting Minds critically reviewed the trial’s methodology, focusing particularly on participant burden and the frequency and duration of survey assessments. The final design—weekly surveys lasting approximately 5−10 minutes over 13 weeks, with a reimbursement of £5 per survey—was deemed acceptable and fair by advisors. Additionally, advisors informed the selection of the control group, strongly advocating for a waitlist control condition. They noted that, although allocation to a waitlist could be initially discouraging for participants, it accurately reflected real-world experiences associated with waiting periods for mental health services. Thus, any dropout observed in this group would realistically represent typical treatment disengagement. As participants in this condition received only our standardized safeguarding procedure (that is, safety planning), it was classified as treatment-as-usual for the purposes of the trial.

Furthermore, Sprouting Minds advisors reviewed and refined participant recruitment materials to ensure clarity and readability for young audiences. Throughout the recruitment phase, advisors actively participated in strategic meetings, providing insights into effective recruitment strategies and the optimal use of various recruitment channels. Upon trial completion, advisors were presented the quantitative analyses of primary and secondary outcomes, with thorough explanations of analytical decisions and processes.

#### Safeguarding

Comprehensive safeguarding measures were implemented, given that study eligibility criteria required participants to have active self-harm ideation. These safeguarding procedures were collaboratively developed with input from Sprouting Minds and drew upon established strategies previously used with youth who self-harm^36,62–66^. Prior to enrollment, participants received detailed information about safeguarding protocols through both the participant information sheet and introductory researcher emails. Participants were required to attend a compulsory 1:1 safety briefing that took place via Zoom, prior to randomization. During briefings, the study purpose, procedures and safeguarding practices were outlined, with participants being invited to ask questions. Participants also engaged in compulsory safety planning.

#### Compulsory safety planning

All participants completed an individualized safety plan^37,38^ before baseline measures and randomization. These briefings were conducted by an ASIST-certified researcher who holds a PhD in suicide research. At the start of a Zoom-based safety briefing, the researcher explained confidentiality, including the circumstances under which emergency services might be contacted (imminent risk of harm to the participant or others), and emphasized that any such action would always be discussed with the participant first. All participants were aged 16 years or older and provided their own informed consent, in line with UK regulations.

Participants were emailed an empty copy of the safety plan using Microsoft Word prior to the video call and were instructed that they could either complete the plan before the call and review it during the briefing or complete it collaboratively with the researcher. Safety planning followed the Stanley−Brown framework^37^, previously adapted and validated for use with LGBTQ+ youth who self-harm^36,62,67^. The template prompted participants to (1) identify internal warning signs (thoughts, feelings and bodily sensations) and external triggers that typically precede a crisis; (2) list social and environmental sources of support; and (3) document relevant professional and crisis services with contact details. Safety plans were sent to participants by email after the briefing, and they were encouraged to retain, update and use these safety plans throughout and beyond the study duration. Additionally, participants nominated a support contact (for example, a parent, a friend over 18 years of age or a general practitioner) who could be contacted if the research team was unable to reach the participant during periods of heightened risk. The nominated contacts received an email to inform them that the participant was taking part in a mental health study and that the research team would be in contact if we were unable to contact the individual after indication of increased risk.

#### Passive safeguarding procedures

Each weekly survey assessment included a visual scale (from 1: ‘very distressed’ to 10: ‘extremely happy’) to evaluate participants’ mood immediately before and after survey completion^66^. Responses were reviewed within 24 hours of survey closure to provide timely feedback on any potential adverse effects of participation, forming one of the triggers for reactive safeguarding (‘Reactive safeguarding procedures’ section). A potential concern was identified when a participant scored lower on the visual scale after survey completion.

At the conclusion of each assessment, participants in both intervention and control groups received contact information about external support services (for example, Kooth, LGBT Foundation Helpline, Young Minds, Samaritans, Mermaids and Allsorts) within Qualtrics and were encouraged to seek professional support if experiencing distress. Such signposting is common practice in mental health research and was effectively used by this research team previously^36,62^.

#### Reactive safeguarding procedures

To prepare for reactive safeguarding, each participant nominated a trusted adult (≥18 years) during the safety planning as a support person whose phone number could be contacted if serious concerns about safety arose; this did not need to be a parent or caregiver, given the potential for non-accepting family contexts. During the study, participants completed weekly assessments that included measures reporting on self-harm, suicidal ideation and mood symptoms. These were used to identify heightened risk during the trial. Responses were reviewed within 24 hours of the survey window closing by an ASIST-trained researcher. A reactive wellbeing check was triggered when participants reported an episode of self-harm in the past week, that they had thoughts of suicide in the past week and if the survey had reduced their mood as identified by the visual scale. This check took place as a phone call between 13:00 and 16:00 the following day. This procedure aligns with established ecological momentary assessment research protocols^62,64^. Across all survey points, 21 reactive wellbeing checks were implemented, and there were no instances where the nominated support person had to be contacted.

Wellbeing calls, in which participants were made aware that researchers were not licensed clinicians, involved empathetic engagement, assessment of current wellbeing (for example, conversations around current mood, recent self-harm thoughts/behaviors and potential suicidal intentions) and explicit advice to seek professional support if needed. Safety plans were reviewed and updated with the young person if needed, to more accurately reflect their experiences and include any further professional services that had been suggested by the researcher. If necessary, the ASIST-trained researcher could contact emergency services with the young person’s consent, mirroring helpline responses^67^. During these wellbeing checks, participants were explicitly reminded that they could withdraw from the study at any time without any penalty and asked if they wished to continue in the study.

If participants were unreachable by phone, researchers sent a follow-up email to check on wellbeing, confirm continued participation and schedule another wellbeing call at a convenient time. If no response was received within 24 hours, another call was made the next day. This process was repeated up to three times. If 4 days passed without a response, the nominated support contact would be notified to ensure the participant’s safety.

### Measures

Data were collected using Qualtrics surveys across 13 weeks, after a prescreening phase used for eligibility and consent, organized into three phases: baseline (weeks 1−3) and intervention deployment (weeks 4−13), of which the follow-up time period was categorized as weeks 11−13. Each survey contained the primary and secondary measures at all timepoints, with three additional measures included at weeks 3, 8 and 13. This decision was made to balance assessment of exploratory outcomes with participant burden. Expectancy measures were not collected, as Purrble was not introduced or described as a therapeutic program or skills-based intervention, and standard expectancy instruments (for example, Credibility/Expectancy Questionnaire^68^) would not have been appropriate in this context. Participants were unfamiliar with Purrble at enrollment and were encouraged simply to explore the device and were not primed to expect specific benefits.

In accordance with the study protocol, outcome measures for the baseline (weeks 1−3) and follow-up (weeks 11−13) phases were calculated as the mean of scores collected during their respective time windows. As the intervention remains embedded within the participant’s environment and is not withdrawn after deployment, participants may continue to engage with it during the follow-up phase in a manner similar to their usage during the active intervention phase. Detailed information regarding the administration schedule for each outcome measure is provided below.

#### Primary outcome: emotion regulation

The DERS-8 (ref. ^69^), an eight-item self-report measure, was used to assess difficulties in emotion regulation. Items were rated on a five-point scale ranging from 1 (almost never (0−10%)) to 5 (almost always (91−100%)). Higher scores indicate greater difficulties in emotion regulation. The DERS-8 was administered at all 13 timepoints (Cronbach’s *α* = 0.87). An example item was: ‘When I’m upset, I have difficulty getting work done’. Previous examinations of the scale have supported its reliability and validity^70^.

### Secondary outcomes

#### Self-harm

The SHQ^71^, comprising three screener items, was used to measure self-harm. As the data were ordinal with unequal intervals and could not be averaged appropriately, each item was examined individually. Items assess self-harm thoughts, suicidal ideation and self-harm behaviors on a frequency scale ranging from 1 (no) to 4 (yes, five or more times)—for example, ‘In the last week, have you thought about harming yourself on purpose, without wanting to die?’ Items were adapted from lifetime prevalence to capture experiences within the last week. Higher scores indicate more frequent occurrences. The SHQ was administered at all 13 timepoints, with self-harm history collected at baseline using the full SHQ^71^. A previous study indicated adequate reliability in adolescents^72,73^.

#### Depression symptoms

Depression symptoms were measured using the PHQ-9 (ref. ^74^), a nine-item measure corresponding to *Diagnostic and Statistical Manual of Mental Disorders*, 4th Edition (DSM-IV) criteria for depression. Participants rated the severity of their depressive symptoms over the past 2 weeks, in which items were rated on a four-point scale from 0 (not at all) to 3 (nearly every day). Higher scores reflect greater depressive severity. The PHQ-9 was assessed at all 13 timepoints (Cronbach’s *α* = 0.86). Previous studies have confirmed the PHQ-9’s reliability and validity^75,76^.

#### Anxiety symptoms

Anxiety symptoms were assessed using the GAD-7 (ref. ^77^), a seven-item measure. Participants rated the severity of their anxiety symptoms over the past week on a four-point scale from 0 (not at all) to 3 (nearly every day). Higher scores represent greater severity of anxiety, with scores of 5, 10 and 15 indicating mild, moderate and severe anxiety, respectively. The GAD-7 was administered at all 13 timepoints (Cronbach’s *α* = 0.87). Previous research has established reliability and validity^75,76^.

### Mechanistic outcomes

#### Hope

Hopefulness was assessed using the State Hope Scale (SHS)^78^. This six-item scale measures goal-directed thinking using two subscales: agency (Cronbach’s *α* = 0.85) and pathways (Cronbach’s *α* = 0.77). The item is scored on an eight-point scale from 1 (definitely false) to 8 (definitely true), with higher scores indicating greater state hope. The SHS was administered at weeks 3, 8 and 13. Previous research has established reliability^79,80^.

#### Loneliness

The UCLA Loneliness Scale (Version 3) (ref. ^81^) was used to measure subjective feelings of loneliness. This scale comprises three questions that measure three dimensions of loneliness—relational connectedness, social connectedness and self-perceived isolation—and was administered at weeks 3, 8 and 13 (Cronbach’s *α* = 0.77). Items are rated between 1 (hardly ever or never) and 3 (often), the total score of which can be categorized into lonely (≥6) or not lonely (≤5). Previous research has established reliability and validity^82,83^.

#### Attentional deployment

The full Process Model Emotion Regulation Questionnaire^84^ is composed of 45 questions to assess uses of 10 emotion regulation strategies. For the present trial, nine questions were used reflecting attentional deployment as an emotion regulation strategy. There are two subscales—focus elsewhere (Cronbach’s *α* = 0.84) and cognitively distract (Cronbach’s *α* = 0.86)—reflecting an engagement and disengagement focus, respectively. To score each subscale, the average of all scale items was calculated. This was administered at weeks 3, 8 and 13*.* Previous research has established reliability and validity^85^.

### Post hoc analyses

#### Perceived Purrble fit/engagement

Fit/engagement with Purrble was assessed for those in the intervention condition using the TWente Engagement with eHealth Technologies Scale (TWEETS)^86^. The TWEETS is a nine-item self-report measure that conceptualizes engagement across behavioral (items 1−3), cognitive (items 4−6) and affective (items 7−9) components. The TWEETS was administered in weeks 4 through 13, after participants had access to Purrble for at least one week. Items were framed as ‘Thinking about using Purrble in the last week…’ and rated on a five-point Likert scale from 0 (strongly disagree) to 4 (strongly agree). Item responses were averaged to yield a total engagement score, with higher scores indicating greater engagement. In this sample, internal consistency was excellent (Cronbach’s *α* = 0.91). The TWEETS has been used with LGBTQ+ youth^36^ and psychometric work found adequate reliability and validity^86^.

### Intervention conditions

Within information sheets and explicitly as part of the compulsory safety briefings, all participants were told that they would be randomly allocated to either intervention (Purrble + SP) or a safety planning plus waitlist condition (SP-Only) as part of the study and that allocation would occur after safety planning and baseline data collection. It was explained that SP-Only participants would receive a Purrble after week 13 of the trial.

#### Safety planning

During the compulsory safety briefing, participants in both conditions engaged in safety planning supported by an ASIST-trained researcher, using the Stanley−Brown framework^37^. Safety plans are a brief collaborative intervention that can help to prevent suicidal behavior^64,87^. The aim of safety plans is to provide participants with a crisis management tool, such that if they are in a period of distress that may lead to self-harm or suicide, they have a physical reminder of their self-harm triggers, potential coping techniques or supports that have previously been helpful and direct contact information for services^88^. Previous research has included safety planning as a standard self-harm safeguarding measure within LGBTQ+ youth research^36,62,67^, enabling participants to identify when they may need to withdraw from a study or seek additional support.

#### Purrble: design and emotion regulation theoretical model

The Purrble intervention consisted of an interactive plush toy (Fig. 1), designed to facilitate immediate soothing responses for participants experiencing emotional distress and to promote long-term improvements in emotion regulation. Full details regarding the toy’s development, design rationale and previous testing were published elsewhere^23,89^.

Participants received Purrble with no instruction of when or how to use the toy, allowing for a discovery process of engagement. They were invited to use Purrble as much or as little as they liked, in whatever capacity suited their needs. Embedded electronics enabled the toy to emit heartbeat-like vibrations, ranging from rapid to slow rhythms. When engaged in specific ways (for example, turning it upside down or touching its ear), the toy initially produces a rapid vibration accompanied by sounds (rapid heartbeat and growling) mimicking signs of anxiety, which slows down as sensors register calming interactions from the participant/user. Sustained soothing results in the toy emitting a steady purring vibration, signaling a calm and contented state. This calming transition typically occurs within 1 minute but could vary depending on participant interaction style.

The theoretical underpinning of the intervention was Gross’ Process Model of Emotion Regulation, which conceptualizes emotion regulation as processes through which individuals influence their emotions, including timing, experience and expression^27^. This theoretical framework informed the following three-level logic model for the intervention.

First, the intervention aimed to provide immediate emotional relief during distressing moments, such as urges to engage in self-harm. Specifically, it is designed to target two stages of emotion regulation: attentional deployment, by redirecting participant focus toward soothing interactions with the toy^28,31,90,91^, and response modulation, facilitating emotional downregulation through tactile engagement analogous to interactions observed in human−animal emotional regulation^92–97^. Second, the intervention sought to promote sustained engagement over time through its conceptual framing. Specifically, participants’ long-term engagement was encouraged by depicting the toy as a vulnerable creature needing care, fostering emotional investment and responsibility similar to long-term interactions with digital pets or social robots^96,97^. Third, repeated use of the intervention aimed to facilitate lasting shifts in participants’ implicit beliefs regarding emotional controllability, thereby promoting healthier emotion regulation practices^44^. Specifically, it was hypothesized to enhance participants’ confidence in their capacity to regulate emotions effectively and reduce reliance on maladaptive strategies such as rumination or suppression.

### Power analysis

A sample size of 70 participants per condition at post-intervention (deployment weeks 11−13) was needed to detect an effect size of 0.4 (Cohen’s *d*) for the primary outcome with a statistical power of (1 − B) = 0.80 in a one-sided *t*-test (*P* = 0.05). A medium effect size of 0.4 was selected as a preliminary exploration based on previous intervention research with and without Purrble^21,42,98–101^, given the uncertainty of the intervention within this population. Therefore, a minimum of 140 participants were needed overall. Considering a predicted dropout rate of 20%, the trial aimed to recruit 168 participants. Because our preregistered analytic plan specified *t*-tests and we subsequently adopted an ANCOVA framework, we examined the effective power of the final sample under the ANCOVA specification. Using Shieh’s exact method for ANCOVA (ANCOVA_analytic in the Superpower package in R) with the observed post-intervention DERS means in the Purrble and waitlist conditions (mean = 25.26 and mean = 28.61, respectively), the within-condition pooled follow-up s.d. = 7.18, three covariates (baseline DERS, age and gender identity) and the empirically estimated covariate *R*^2^ = 0.38, the achieved sample size yielded an estimated power of 91.7% to detect the observed emotion regulation effect at *α* = 0.05.

### Data statistical analysis

Analyses were conducted using R statistical software. We first conducted preliminary analyses, including basic descriptive analysis with skewness and kurtosis to assess normality. All statistical tests were two-sided. We assessed equivalence of demographic (age only) and baseline variables across conditions using *t*-tests for continuous variables and *χ*^2^ tests for categorical variables. We did not test equivalence for sexual identity or race/ethnicity due to the small sample size, the high number of categories in each demographic variable and low membership within most categories. Equivalence by gender identity was not tested, as randomization procedures already accounted for gender identity. Second, we performed multivariate outlier analyses to identify influential data points^102^. Third, we conducted attrition analyses^103^, with attrition operationalized as participants failing to fill in any follow-up questionnaires (weeks 11−13). A binary indicator was created to represent follow-up completion (1 = filled in at least one follow-up questionnaire; 0 = filled in none). Attrition rates were calculated overall, by condition and by gender identity, using *χ*^2^ tests to determine whether attrition differed by condition or gender identity. Then, to assess potential attrition bias, we conducted two-way ANOVAs testing for condition × attrition status effects on each baseline outcome variable (except for self-harm; see below).

#### Deviations from preregistered analysis plan

The preregistered protocol specified one-tailed paired *t*-tests to evaluate intervention effects by comparing averaged baseline and follow-up scores. Prior to analysis, the team decided, instead, to use two-tailed ANCOVA models (adjusting for baseline scores, age and gender identity) as the primary approach. This change reflected (1) recognition of the possibility of bidirectional effects, including potential iatrogenic effects, and, therefore, the need for two-sided hypothesis testing, and (2) the greater statistical rigor of ANCOVA in controlling for baseline levels of the outcome, improving precision and accounting for any small group imbalances. The protocol indicated that covariates would be included but did not specify which ones or how they would be selected; we, therefore, prespecified age (to capture potential differences across the 16−25-year age range) and gender identity (cisgender versus TGD) as covariates.

The original protocol did not outline moderator analyses. After preregistration, and after consultation with an independent statistician, we expanded the analytic plan to test whether gender identity moderated intervention effects. This addition was motivated by concerns in the LGBTQ+ literature about treating TGD youth as homogeneous with sexual minority youth, thereby obscuring potentially distinct patterns of response.

The protocol stated that we would examine moderation by engagement for the three clinical outcomes (self-harm, depression and anxiety). These engagement analyses were not included in the present paper; instead, we plan to report them in a separate paper focused specifically on engagement processes in the future.

For self-harm outcomes, the protocol implied that the same analytic approach (*t*-tests or analogous methods) would be used for all outcomes, presumably averaging weeks 1−3 and weeks 11−13. In practice, because the self-harm variables were assessed on a four-point ordinal scale with unequal intervals, this was not possible. As such, we analyzed week 1 versus week 12 only and used ordinal logistic regression rather than *t*-tests or ANCOVA, based on discussions between the independent statistician and self-harm experts on the team regarding the most appropriate and interpretable approach.

Finally, although the original document suggested including baseline DERS-8 as a covariate in secondary analyses, we did not adjust for baseline DERS-8 in models predicting depression, anxiety or self-harm. This decision, informed by statistical advice and relevant literature^104^, reflected concerns that adjusting for emotion regulation, given its close conceptual and empirical relation to these clinical outcomes, could overcontrol and bias estimates toward the null.

#### Primary and secondary analyses

As outlined in the preregistration, participant scores were averaged across weeks 1−3 to compute their baseline scores and across weeks 11−13 to compute their follow-up scores. The analytic approach specified in the preregistration originally indicated that intervention effects would be evaluated using one-tailed paired *t*-tests. However, upon consultation with an independent statistician who joined the research team, this strategy was revised prior to data analysis. The primary analyses were modified to use two-tailed ANCOVA instead of one-tailed paired *t*-tests. This adjustment addressed methodological concerns regarding the potential for bidirectional intervention effects, as the theoretical framework did not explicitly exclude the possibility of iatrogenic effects. Additionally, ANCOVA allowed for statistical control of baseline outcome scores, enhancing precision in estimating intervention efficacy. This method also facilitated the inclusion of theoretically relevant covariates known to introduce variability within the target age range (16−25 years). Specifically, age was included due to its developmental importance, given the considerable cognitive, emotional and social changes occurring during this period, along with related variability in support structures (for example, living situations and educational contexts). Second, gender identity was included as a covariate due to the heightened risk of poor adverse outcomes among TGD youth compared to sexual orientation-only youth^40,105^ alongside unique, additional experiences (for example, gender dysphoria, transphobia and difficulties with transitioning)^106^.

For all main effects analyses except self-harm, we examined individual-level change using reliable change indices (RCIs) for emotion regulation difficulties (DERS-8), anxiety (GAD-7) and depressive symptoms (PHQ-9)^107^. For each scale, we used the baseline standard deviation and its internal consistency (Cronbach’s *α*) as the reliability estimate to derive an RCI score for each participant, and we classified outcomes as reliable improvement, reliable deterioration, or no reliable change based on the ±1.96 criterion. We then compared conditions using odds ratios with 95% CIs, estimating (1) the odds of reliable improvement versus all other outcomes and (2) the odds of reliable deterioration versus all other outcomes for Purrble plus safety planning relative to safety planning alone.

#### Exploratory analyses

Furthermore, for all main effects analyses except self-harm in addition to the main ANCOVA models, we conducted parallel linear mixed models (LMMs) for each outcome to examine changes across the weekly assessments.

#### Post hoc analyses

After registration, the analytic plan was expanded to explore potential moderation effects. Specifically, we introduced secondary post hoc interaction analyses examining whether gender identity (cisgender versus TGD) moderated intervention effectiveness. Given the lack of previous research, no a priori hypotheses were made for the gender identity moderation analyses.

#### Self-harm analyses

Each self-harm variable was measured on a four-point ordinal scale with unequal intervals. As such, two changes from the preregistered protocol had to be made. First, we did not create averages of ‘baseline’ and ‘follow-up’ for the first weeks given that the unequal intervals would make averages difficult to interpret. As such, we selected week 1 as our baseline week (the first week and the week with the largest sample size) and week 12 (the ‘middle’ of the follow-up week with at least 2 weeks past the intervention) as our follow-up week for self-harm variables. Furthermore, given the unequal intervals, the data did not meet the baseline assumptions necessary for conducting *t*-tests or ANCOVA. Given the structure of the data, we considered two potential approaches: ordinal logistic regression using, or odds ratios using collapsed binary versions to indicate presence versus absence of self-harm. In ordinal logistic regression models, the relation between predictors and the outcome is assumed to be homogeneous across the outcome’s ordinal level. There are two ways in which this could be conceptualized. First, statistically—meaning that variables can be tested using Brant’s test to see if they violate the proportional odds assumption. Second, theoretically—in other words: Does the idea of equal slopes across variables make sense in theory given the nature of the content (that is, moving from no self-harm at all to some level of self-harm being equivalent to moving from some self-harm to higher levels of self-harm, for example)? We considered that, from a theoretical perspective, there may be a clinical difference between change from one active harm category to another (reduction or increase) and change between a total absence of self-harm to presence of harm. As such, we opted to take the conservative approach of using a recommended conservative estimate for the Brant test (*P* > 0.2) for the proportional odds assumption^108^. If all variables included in the models (week 1 and week 12) passed the assumption, we would use the proportional odds regression. Otherwise, we would collapse the models into binary categories—specifically, using ‘no self-harm’ coded as 0 versus ‘any self-harm’ coded as 1.

The results of the Brant tests revealed that all week 1 and week 12 had *P* > 0.2. As such, we opted to use ordinal logistic regression models without dichotomizing the outcome. We conducted preliminary analyses including attrition analyses and baseline equivalence tests (using the non-parametric Mann−Whitney *U*-test). Frequencies were examined to understand data distributions. We then used ordinal logistic regression to examine intervention outcomes, controlling for age and gender identity. Finally, we examined the interaction of condition with baseline self-harm and gender identity on the outcome.

### Reporting summary

Further information on research design is available in the Nature Portfolio Reporting Summary linked to this article.

## Online content

Any methods, additional references, Nature Portfolio reporting summaries, source data, extended data, supplementary information, acknowledgements, peer review information; details of author contributions and competing interests; and statements of data and code availability are available at 10.1038/s41591-026-04422-6.

## Supplementary information

Supplementary Information Consortia members

Reporting Summary

Peer Review File

## Figures and Tables

**Extended Data Table 1:**
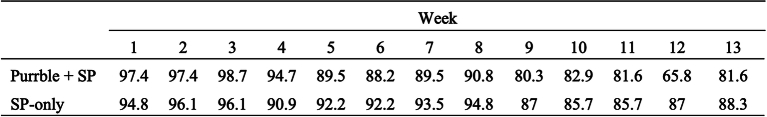
Weekly survey response rates (% of total randomised) by condition

*Note*. Values represent the percentage of all randomised participants in each condition who contributed usable survey data in each study week. Partial responses were included if they yielded at least one interpretable score; operationally, this required sufficient completion of the first weekly measure (DERS-8) to be scored.

**Extended Data Table 2:**
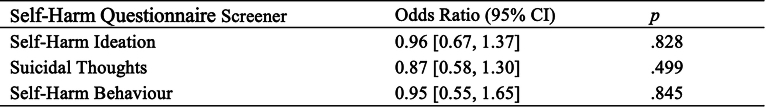
Proportional odds logistic regression models predicting week-12 self-harm category from intervention condition

*Note*. Each model is a proportional odds logistic regression of the Week-12 screener outcome on condition (Purrble + SP vs SP-only). All models controlled for the corresponding baseline screener score (Week 1), age, and gender identity and tested, separately, whether baseline score or gender identity moderated the condition effect; no interactions reached significance (all p > .05). Brant’s tests confirmed that the proportional-odds assumption was met for every model (all p > .05). No corrections were made for multiple comparisons.

**Extended Data Table 3:**
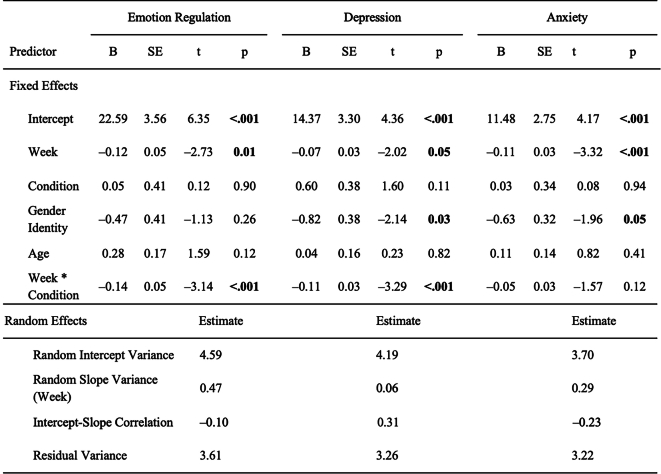
Linear mixed-effects models (LMMs) of symptom trajectories over time by condition

*Note*. Results are from separate two-sided linear mixed-effects models with random intercepts and random slopes for Week, estimating symptom trajectories from Week 0 to Week 13. Fixed effects included Week, condition, gender identity, age, and the Week × Condition interaction. Week was coded 0–13. Condition was coded 0 = SP-only and 1 = Purrble + SP. Gender identity was coded 0 = cisgender and 1 = transgender/gender diverse. Accordingly, the Week coefficient represents the weekly rate of change in the SP-only group, and the Week × Condition term tests whether the rate of change differed in the Purrble + SP group. B = unstandardised regression coefficient. No correction for multiple comparisons was applied. Bolded coefficients indicate p < .05.

**Extended Data Table 4:**
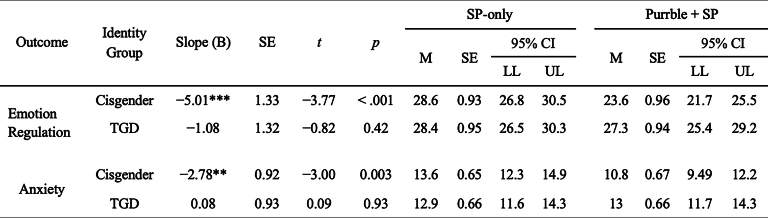
Slopes and estimated marginal means for significant condition × gender identity interactions

*Note*. Results are from two-sided ANCOVA models estimated using linear regression, with post-intervention outcome regressed on condition, baseline outcome, gender identity, and age. Gender Identity coded 0 = cisgender, 1 = transgender/gender diverse. Condition coded 0 = Safety Planning Only, 1 = Purrble + Safety Planning. Simple slopes represent the conditional effect of intervention condition within each gender identity group. Estimated marginal means represent follow-up means by intervention for each identity group, and were obtained from the same fitted models using emmeans, with continuous covariates held at their sample means. B = unstandardised regression coefficient; CI = confidence interval; LL = lower limit; UL = upper limit; TGD = transgender/gender diverse. The test statistic reported for each simple slope is *t* with 134 residual degrees of freedom for all models. No corrections were made for multiple comparisons. *p* < .05*, *p* < .01**, *p* < .001***.

**Extended Data Table 5:**
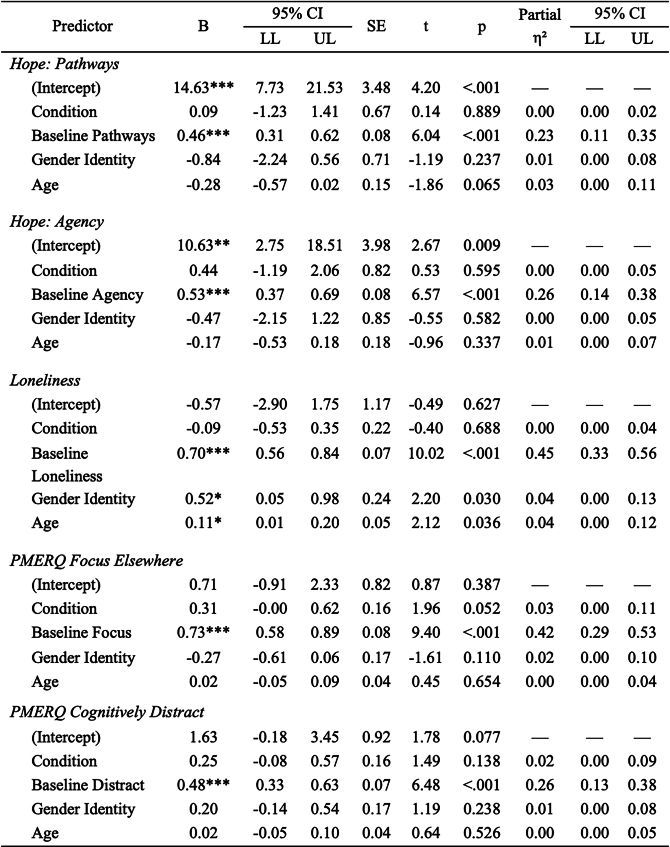
Purrble effects on mechanistic outcomes: hope, loneliness and attentional deployment

*Note*. Results are from two-sided ANCOVA models estimated using linear regression, with post-intervention outcome regressed on condition, baseline outcome, gender identity, and age. Gender Identity coded 0 = cisgender, 1 = transgender/gender diverse. Condition coded 0 = Safety Planning Only, 1 = Purrble +Safety Planning. B = unstandardised regression coefficient. CI = confidence interval. LL = lower limit; UL = upper limit. Partial η^2^ reflects effect size for each predictor. *p* < .05*, *p* < .01**, *p* < .001***. The test statistic reported for each coefficient is *t*, with residual degrees of freedom of 121 for the Hope Pathways, Hope Agency, and Loneliness models, and 120 for the PMERQ models. No corrections for multiple comparisons were made.

**Extended Data Table 6:**
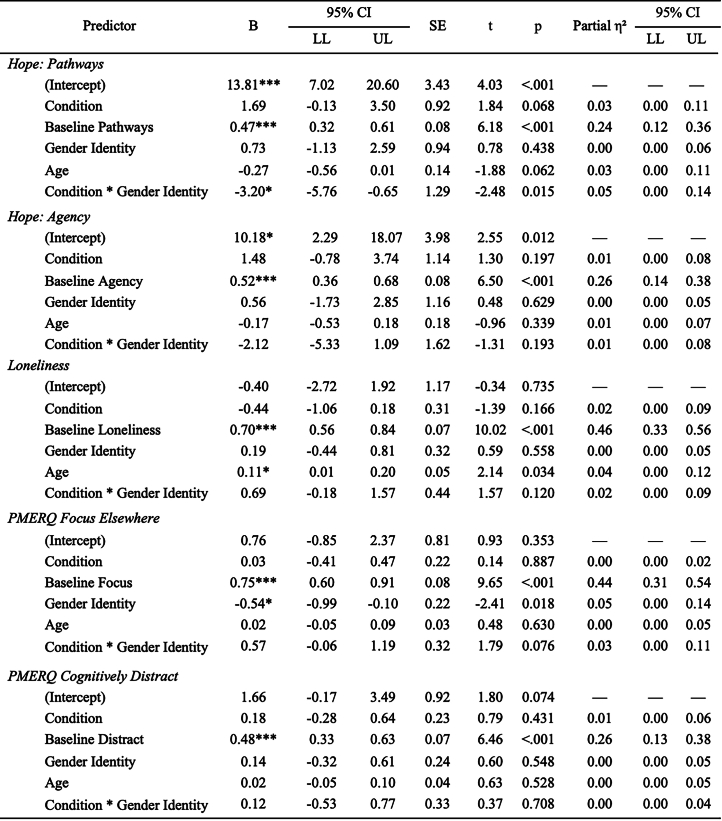
Gender identity by condition interactive effects on mechanistic outcomes

*Note*. Results are from two-sided ANCOVA models estimated using linear regression, with post-intervention outcome regressed on condition, baseline outcome, gender identity, age, and the Condition × Gender Identity interaction. Gender Identity coded 0 = cisgender, 1 = transgender/gender diverse. Condition coded 0 = Safety Planning Only, 1 = Purrble +Safety Planning. B = unstandardised regression coefficient. CI = confidence interval. LL = lower limit; UL = upper limit. Partial η^2^ reflects effect size for each predictor. *p* < .05*, *p* < .01**, *p* < .001***. The test statistic reported for each coefficient is *t*, with residual degrees of freedom of 120 for the Hope Pathways, Hope Agency, and Loneliness models, and 119 for the PMERQ models. No corrections for multiple comparisons were made.

## Data Availability

Because the intervention involved a physical, visually recognizable device (Purrble), and because recruitment occurred within a limited geographic area and defined time window, there is an increased risk that individuals familiar with participants could infer trial participation (for example, by having seen a participant with the device) when combined with other potentially identifying information. This risk is further amplified by the inclusion of sensitive self-harm-related outcomes. In combination with demographic variables such as age, gender identity, sexuality or race, these factors create a meaningful risk of participant reidentification, even after removal of direct identifiers. To balance transparency with participant protection, a minimally deidentified dataset that excludes all demographic variables and other information that could substantially contribute to reidentification has been made publicly available in figshare at 10.6084/m9.figshare.31859155. Researchers who would like to replicate the full set of analyses or access the fully anonymized analytic dataset may submit a request for materials. Requests should be addressed to P.S. (petr.slovak@kcl.ac.uk). Access to the full dataset will require execution of a data use agreement between the requesting party and the data holder (King’s College London) that prohibits attempts at reidentification and specifies appropriate data security and storage procedures, consistent with the study’s ethical approvals. Requests will be reviewed for alignment with these requirements by the King’s College London legal team, with any approvals not unreasonably withheld. Once approved, data would normally be provided within 1 month of agreement completion.

## Code availability

The code for the statistical analysis is available from C.A.R. and has been uploaded to GitHub (https://github.com/caarhodes/Purrble).
