# Supplementary material for: A socially assistive robot to support mental wellbeing in LGBTQ+ young people at risk of self-harm: a randomized controlled trial
Source: Nat Med. 2026 Jun 4;32(6):2267–76. doi: 10.1038/s41591-026-04422-6 (PMC13279257; doi:10.1038/s41591-026-04422-6)
Supplement: Supplementary file 1 — Consortia members [file 41591_2026_4422_MOESM1_ESM.pdf]

nature medicine

Supplementary information https://doi.org/10.1038/s41591-026-04422-6

A socially assistive robot to support mental wellbeing in LGBTQ+ young people at risk of self-harm: a randomized controlled trial

In the format provided by the authors and unedited

Digital Youth research team

Petr Slovak[1], A. Jess Williams[1,2,3], Chris Hollis[12,13], Ellen Townsend[3,14], Jo Gregory[15], Rebecca Woodcock[15], Sarah Doherty[15], Lucy-Paige Willingham[15], Emma Nielsen[15], Elvira Perez Vallejos[15], Louise Arseneault[16], Peter Fonagy[17], Cathy Creswell[18], Emily Lloyd[18], Josimar De Alcantara Mendes[19], Carolyn Ten Holter[19], Marina Jirotka[19], Praveetha Patalay[17], Yvonne Kelly[17], Aaron Kandola[17], Yi Yang[17], Edmund Sonuga-Barke[16], Sonia Livingstone[20], Kasia Kostryka-Allchorne[21], Jake Bourgaize[16], Mariya Stoilova[20], Aja Murray[22], Peiyao Tang[16], Rory O'Connor[4], Dorothee Auer[15], Sieun Lee[15], Nitish Jawahar[15], Marianne Etherson[4], Chris Greenhalgh[15], Kapil Sayal[15], Jim Warren[23], Vajisha Wanniarachchi[23], Kevin Glover[15], Paul Stallard[24], Charlotte Hall[15], Mathijs Lucassen[25], Sally Merry[23], Karolina Stasiak[23], Camilla Babbage[15], Adam Parker[15], Holly Griffiths[15], Lily Roberts[15], Joanna Lockwood[15], Lucy Hitcham[15].

Affiliations

[1] Department of Informatics, King's College London, London, UK
[2] National Centre for Suicide Prevention and Self-Harm Research, Swansea University Medical School, Swansea, UK
[3] Institute of Mental Health, University of Nottingham, Nottingham, UK
[4] University of Glasgow, Glasgow, UK
[5] Health Service and Population Research Department; Institute of Psychiatry, Psychology & Neuroscience; King's College London; London, UK
[6] Department of Psychiatry, University of Oxford, Oxford, UK
[7] Oxford Health NHS Foundation Trust, Oxfordshire, UK
[8] Justice Health Group; School of Population Health, Curtin University, Perth, Australia
[9] Centre for Adolescent Health, Murdoch Children's Research Institute, Royal Children's Hospital, Melbourne, Australia
[10] Department of Psychology, Stanford University, Stanford, California, USA
[11] Division of Clinical Neuroscience, University of Nottingham, Nottingham, UK
[12] National Institute of Health Research (NIHR) MindTech MedTech Research Centre, Institute of Mental Health, School of Medicine, University of Nottingham, Nottingham, United Kingdom
[13] Nottinghamshire Healthcare NHS Foundation Trust, Nottingham, UK
[14] School of Psychology, University of Nottingham, Nottingham, UK
[15] University of Nottingham, Nottingham, UK
[16] King's College London, London, UK
[17] University College London, London, UK
[18] Department of Experimental Psychology, University of Oxford, Oxford, UK
[19] Department of Computer Science, University of Oxford, Oxford, UK
[20] London School of Economics and Political Science, London, UK
[21] Queen Mary University of London, London, UK
[22] University of Edinburgh, Edinburgh, UK
[23] University of Auckland, Auckland, New Zealand
[24] University of Bath, Bath, UK
[25] City St. George's, University of London, London, UK
