# Supplementary material for: A socially assistive robot to support mental wellbeing in LGBTQ+ young people at risk of self-harm: a randomized controlled trial
Source: Nat Med. 2026 Jun 4;32(6):2267–76. doi: 10.1038/s41591-026-04422-6 (PMC13279257; doi:10.1038/s41591-026-04422-6)
Supplement: Supplementary file 2 — Reporting Summary [file 41591_2026_4422_MOESM2_ESM.pdf]

nature portfolio

Corresponding author(s): Petr Slovak
Last updated by author(s): 01/04/2026

Reporting Summary

Nature Portfolio wishes to improve the reproducibility of the work that we publish. This form provides structure for consistency and transparency in reporting. For further information on Nature Portfolio policies, see our Editorial Policies and the Editorial Policy Checklist.

Statistics

For all statistical analyses, confirm that the following items are present in the figure legend, table legend, main text, or Methods section.

n/a	Confirmed	
☐	☒	The exact sample size (*n*) for each experimental group/condition, given as a discrete number and unit of measurement
☐	☒	A statement on whether measurements were taken from distinct samples or whether the same sample was measured repeatedly
☐	☒	The statistical test(s) used AND whether they are one- or two-sided<br>*Only common tests should be described solely by name; describe more complex techniques in the Methods section.*
☐	☒	A description of all covariates tested
☐	☒	A description of any assumptions or corrections, such as tests of normality and adjustment for multiple comparisons
☐	☒	A full description of the statistical parameters including central tendency (e.g. means) or other basic estimates (e.g. regression coefficient) AND variation (e.g. standard deviation) or associated estimates of uncertainty (e.g. confidence intervals)
☐	☒	For null hypothesis testing, the test statistic (e.g. *F*, *t*, *r*) with confidence intervals, effect sizes, degrees of freedom and *P* value noted<br>*Give P values as exact values whenever suitable.*
☒	☐	For Bayesian analysis, information on the choice of priors and Markov chain Monte Carlo settings
☒	☐	For hierarchical and complex designs, identification of the appropriate level for tests and full reporting of outcomes
☐	☒	Estimates of effect sizes (e.g. Cohen's *d*, Pearson's *r*), indicating how they were calculated

Our web collection on statistics for biologists contains articles on many of the points above.

Software and code

Policy information about availability of computer code

Data collection	Data were collected using Qualtrics survey software. No custom code was used for data collection.
Data analysis	Data were analysed in R using RStudio (version 2024.09.1+394, Posit Software, PBC). Key R packages used for the reported analyses included dplyr and tidyr for data processing, psych for descriptive statistics, car for regression/ANCOVA-related tests, effectsize for effect-size estimation, and lme4 and emmeans for linear mixed-effects modelling and post hoc estimated marginal means, where applicable. No custom algorithms were used.

For manuscripts utilizing custom algorithms or software that are central to the research but not yet described in published literature, software must be made available to editors and reviewers. We strongly encourage code deposition in a community repository (e.g. GitHub). See the Nature Portfolio guidelines for submitting code & software for further information.

Data

Policy information about availability of data

All manuscripts must include a data availability statement. This statement should provide the following information, where applicable:

- Accession codes, unique identifiers, or web links for publicly available datasets
- A description of any restrictions on data availability
- For clinical datasets or third party data, please ensure that the statement adheres to our policy

Because the intervention involved a physical, visually recognisable device (Purrble), and because recruitment occurred within a limited geographic area and defined

time window, there is an increased risk that individuals familiar with participants could infer trial participation (for example, by having seen a participant with the device) when combined with other potentially identifying information. This risk is further amplified by the inclusion of sensitive self-harm–related outcomes. In combination with demographic variables such as age, gender identity, sexuality, or race, these factors create a meaningful risk of participant re-identification, even after removal of direct identifiers.
To balance transparency with participant protection, a minimally deidentified dataset that excludes all demographic variables and other information that could substantially contribute to re-identification has been are publicly available in Figshare at https://doi.org/10.6084/m9.figshare.31859155.
Researchers who wish to replicate the full set of analyses or access the fully anonymised analytic dataset may submit a request for materials. Requests should be addressed to Dr. Petr Slovak (petr.slovak@kcl.ac.uk). Access to the full dataset will require execution of a data use agreement between the requesting party and data holder (King's College London) that prohibits attempts at re-identification and specifies appropriate data security and storage procedures, consistent with the study's ethical approvals. Requests will be reviewed for alignment with these requirements by KCL legal team, with any approvals not unreasonably withheld. Once approved, data would normally be provided within one month of agreement completion.

Research involving human participants, their data, or biological material

Policy information about studies with human participants or human data. See also policy information about sex, gender (identity/presentation), and sexual orientation and race, ethnicity and racism.

Reporting on sex and gender	The study focuses on LGBTQ+ young people, therefore we have collected self-reported gender and not collected biological sex or sex assigned at birth.
Reporting on race, ethnicity, or other socially relevant groupings	Socially constructed or socially relevant variables used in the manuscript included sexual orientation/gender identity minority status, gender identity, race/ethnicity, and current education level/occupation. Eligibility for the study required that participants identify as a sexual orientation and/or gender identity minority (LGBTQ+). Demographic information was provided directly by participants through self-report at study entry. Race/ethnicity and current education level/occupation were collected using open-text self-report and, for descriptive reporting only, were simplified into broader categories. These simplified categories were used to describe the study sample and were not used as proxies for other socially constructed variables such as socioeconomic status. Gender identity was also self-reported and was used in adjusted analyses where specified in the manuscript. Because participants were enrolled on the basis of LGBTQ+ identity, this characteristic reflects the target population rather than a comparison variable. Confounding was addressed primarily through randomization and, in adjusted outcome analyses, by including prespecified covariates such as baseline outcome values, age, and gender identity.
Population characteristics	Participants were adolescents and young adults aged 16–25 years living in the United Kingdom who identified as a sexual orientation and/or gender identity minority (LGBTQ+), reported recent self-harm ideation within the past month, and were able to read and speak English. Participants also completed baseline demographic questions, including age, gender identity, race/ethnicity, and current education level/occupation, via self-report. These characteristics were used to describe the sample, and selected variables were included as covariates in adjusted analyses as specified in the manuscript.
Recruitment	Participant recruitment occurred between January and September 2024 and used a multi-channel strategy to reach eligible young people. Recruitment methods included online platforms (Twitter/X, Instagram, paid research participation sites, university participant pools, and MQ Mental Health Research), physical community outreach (for example, schools and community centres), and snowball sampling methods (including word of mouth and the Volunteer Tutors Organization). Interested individuals registered their interest and were screened for eligibility. As with many studies using online and community-based recruitment, there is potential for self-selection bias. In particular, because the study was advertised using images of Purrble, participants may have been more likely to enrol if they were already positively inclined toward comforting, plush, or socially assistive robotic companions. This may have influenced the findings if participants with a pre-existing interest in such technologies were more receptive to the intervention or more likely to perceive it positively. These factors may affect the representativeness of the sample, although randomisation helps reduce bias in comparisons between study arms.
Ethics oversight	All study procedures were reviewed and approved by the King's College London research ethics committee (RESCM-22/23-34570). The study was conducted under ethics committee oversight, and all participants provided informed consent before participation.

Note that full information on the approval of the study protocol must also be provided in the manuscript.

Field-specific reporting

Please select the one below that is the best fit for your research. If you are not sure, read the appropriate sections before making your selection.

☐ Life sciences ☒ Behavioural & social sciences ☐ Ecological, evolutionary & environmental sciences

For a reference copy of the document with all sections, see nature.com/documents/nr-reporting-summary-flat.pdf

Behavioural & social sciences study design

All studies must disclose on these points even when the disclosure is negative.

Study description	This study was a quantitative, single-blind, longitudinal, parallel, stratified randomized controlled trial conducted with LGBTQ+ young people in the UK. Participants completed repeated self-report assessments across a 13-week trial. After a 3-week baseline period,

eligible participants were randomized 1:1 to either Purrble plus safety planning or safety planning plus waitlist control, with randomization stratified by gender identity (cisgender vs transgender and gender-diverse).

Research sample	The research sample comprised LGBTQ+ young people aged 16–25 years living in the UK who reported self-harm ideation or behaviour within the previous month. Eligibility criteria required participants to be aged 16–25 years inclusive, reside in the UK for the duration of the study, identify as a sexual orientation and/or gender identity minority, report recent self-harm ideation or behaviour, and be proficient in reading, writing, and speaking English. Individuals were excluded if they identified as cisgender and heterosexual, were outside the age range, did not report recent self-harm ideation or behaviour, or lived outside the UK. This sample was chosen because LGBTQ+ young people with recent self-harm thoughts or behaviours represent a population with high and often immediate mental health support needs, while also facing barriers to accessing appropriate services. We used a wide range of recruitment methods to maximize diversity of outreach and to approach the broadest feasible representation of eligible young people. Demographic information, including age, gender identity, race/ethnicity, and current education level/occupation, was collected by self-report at baseline.
Sampling strategy	Participants were recruited using a non-probability, multi-channel sampling strategy that combined convenience and snowball sampling methods. Recruitment occurred through online platforms (Twitter/X, Instagram, paid research participation sites, university participant pools, and MQ Mental Health Research), physical community outreach (e.g., schools and community centres), and snowball methods (e.g., word of mouth and the Volunteer Tutors Organization). This approach was used to maximize reach to eligible young people in the target population. Recruitment was discontinued in September 2024 after the target minimum sample size had been achieved and registration rates had substantially decreased, suggesting saturation of available recruitment channels. Because recruitment relied on convenience and snowball methods rather than probability sampling, the sample may not be representative of the wider population of LGBTQ+ young people with recent self-harm ideation or behaviour.
Data collection	Data were collected remotely using Qualtrics surveys and Zoom-based intake procedures. Interested individuals first completed pre-screening for eligibility and consent procedures. Before enrolment into the trial, participants completed a compulsory 1:1 synchronous safety briefing and safety planning session via Zoom with an ASIST-trained researcher in a private office space; participants were asked to join the call from a private, quiet location of their choice. After completion of baseline procedures and the Week 3 assessment window, participants were randomized and then completed weekly online self-report surveys hosted in Qualtrics for the 13-week trial. Surveys were emailed to participants, followed by a next-day automatic reminder from Qualtrics and a personalized reminder message for non-completion the following day. Surveys remained open for 3 days. Data collection was standardized across study arms. Participants completed surveys independently online, without the researcher present during survey completion.
Timing	Participant recruitment occurred between January 2024 and September 2024. The trial lasted 13 weeks per participant, with the first 3 weeks constituting baseline assessment, followed by randomization and a 10-week deployment period. Extended assessments were conducted at Week 3, Week 8, and Week 13. Final follow-up assessments were completed in October 2024.
Data exclusions	Two individuals who had been randomized were excluded from the final analytic dataset because they did not contribute trial data: one withdrew before trial data collection began and one could not be verified before trial data collection began. A final exclusion was made during data analysis, due to filing errors that took place during the data collection process.
Non-participation	Two randomized individuals did not participate in the trial data collection period. One withdrew before data collection began and one could not be verified.
Randomization	Randomization was conducted after consent and baseline data collection by the study coordinator, who was the only person with access to the randomization process. Participants were grouped into blocks based on similar enrolment timeframes and then individually randomized within block in a 1:1 ratio to Purrble plus safety planning or safety planning plus waitlist control. Allocation was stratified by gender identity (cisgender vs transgender and gender-diverse) to promote balance across study arms and was implemented using an online randomization generator. Participants were informed of their allocation by email after closure of the Week 3 data collection window.

Reporting for specific materials, systems and methods

We require information from authors about some types of materials, experimental systems and methods used in many studies. Here, indicate whether each material, system or method listed is relevant to your study. If you are not sure if a list item applies to your research, read the appropriate section before selecting a response.

Materials & experimental systems

n/a	Involved in the study	
☒	☐	Antibodies
☒	☐	Eukaryotic cell lines
☒	☐	Palaeontology and archaeology
☒	☐	Animals and other organisms
☐	☒	Clinical data
☒	☐	Dual use research of concern
☒	☐	Plants

Methods

n/a	Involved in the study	
☒	☐	ChIP-seq
☒	☐	Flow cytometry
☒	☐	MRI-based neuroimaging

Clinical data

Policy information about clinical studies

All manuscripts should comply with the ICMJE guidelines for publication of clinical research and a completed CONSORT checklist must be included with all submissions.

Clinical trial registration	ClinicalTrials.gov: NCT06025942
Study protocol	https://doi.org/10.1136/bmjopen-2023-079801
Data collection	All data were collected online. Recruitment period: 12th January - 1st September 2024. All data was collected by 22nd of October 2024.
Outcomes	The primary outcome was emotion regulation, assessed using the DERS-8. Secondary outcomes were self-harm, depression, and anxiety, assessed using the SHQ (items analysed separately), PHQ-9, and GAD-7, respectively. Mechanistic outcomes were loneliness, hope, and attentional deployment, assessed using the UCLA Loneliness Scale, the State Hope Scale (agency and pathways subscales), and PMERQ attentional deployment subscales (focus elsewhere and cognitively distract). Primary and secondary outcomes were assessed at all 13 timepoints, whereas mechanistic outcomes were assessed at Weeks 3, 8, and 13. Baseline and follow-up values were calculated as the mean of scores across the relevant assessment windows, as pre-specified in the protocol.

Plants

Seed stocks	*Report on the source of all seed stocks or other plant material used. If applicable, state the seed stock centre and catalogue number. If plant specimens were collected from the field, describe the collection location, date and sampling procedures.*
Novel plant genotypes	*Describe the methods by which all novel plant genotypes were produced. This includes those generated by transgenic approaches, gene editing, chemical/radiation-based mutagenesis and hybridization. For transgenic lines, describe the transformation method, the number of independent lines analyzed and the generation upon which experiments were performed. For gene-edited lines, describe the editor used, the endogenous sequence targeted for editing, the targeting guide RNA sequence (if applicable) and how the editor was applied.*
Authentication	*Describe any authentication procedures for each seed stock used or novel genotype generated. Describe any experiments used to assess the effect of a mutation and, where applicable, how potential secondary effects (e.g. second site T-DNA insertions, mosiacism, off-target gene editing) were examined.*
