# Supplementary material for: A socially assistive robot to support mental wellbeing in LGBTQ+ young people at risk of self-harm: a randomized controlled trial
Source: Nat Med. 2026 Jun 4;32(6):2267–76. doi: 10.1038/s41591-026-04422-6 (PMC13279257; doi:10.1038/s41591-026-04422-6)
Supplement: Supplementary file 3 — Peer Review File [file 41591_2026_4422_MOESM3_ESM.pdf]

nature portfolio

Peer Review File

A socially-assistive robot to support mental wellbeing in LGBTQ+ young people at risk of self-harm: a randomised controlled trial

Corresponding Author: Dr Petr Slovak

This file contains all reviewer reports in order by version, followed by all author rebuttals in order by version.

Version 1:

Reviewer comments:

Reviewer #1

(Remarks to the Author)

Summary of the key results
This pre-registered, parallel-group RCT evaluated the socially assistive robot Purrble for LGBTQ+ youth (16–25 years) with recent self-harm ideation, randomised to intervention vs. waitlist for 13 weeks. The primary endpoint (emotion regulation, DERS-8) improved signifi-cantly in the intervention arm ($\eta^2p = 0.09$), with additional benefits observed on GAD-7 and PHQ-9, particularly among cisgender participants. No effect was detected on self-harm. Re-tention was high, though weekly survey completion declined faster in the intervention arm.
Overall, the study is reported in line with the published protocol.
In general, the study design (intervention vs. waitlist, unblinded self-report outcomes) limits the strength of the conclusions. Participants knew allocation, the waitlist would receive Purrble after week 13, and all participants could keep devices. Together with £5 per survey, this may have amplified expectancy and engagement differences. Waitlist designs are vulnerable to inflated effects on self-report outcomes. Claims should therefore be tempered and framed as provisional until replicated with an active or placebo control. If expectancy measures were collected, these should be adjusted for or, if not, the absence should be discussed.
Originality and significance
The intervention is novel in targeting emotion regulation through a socially assistive robot in a high-risk LGBTQ+ population. This is an important and underserved group. To my knowledge, few if any RCTs have tested similar interventions in this setting. The work has the potential to be clinically meaningful if replicated with more rigorous controls.
The introduction is somewhat superficial. Please situate the work more clearly within prior literature: have other innovative interventions been trialed in this population, and how does this study extend or deviate from existing approaches?
Data & methodology
The study has several methodological strengths: prospective registration, publication of a pro-tocol, transparent reporting of deviations, a clear safeguarding plan, and strong participant retention. However, there are limitations:
Ensure all abbreviations are introduced (e.g. “TGD” appears in the Results without definition).
Please justify the age range (16–25).
Clarify whether a cut-off for self-harm ideation severity was required for inclusion, as this af-fects generalizability.
Report the response rate to weekly surveys over time. Declining engagement is common in mental health populations and raises risk of selective reporting.
Provide details on the qualitative process analyses: how many participants were included, what were their characteristics, and do they reflect the overall sample?
Confirm whether a statistical analysis plan was published in addition to the protocol.
Randomisation requires fuller reporting: how was the sequence generated, was blocking or stratification used (beyond gender identity), who had access to the sequence, and what sa-feguards prevented foreknowledge or manipulation? Was allocation performed after consent and baseline assessments?
Outcomes appear to rely exclusively on self-report. Without blinded observer-rated measures, and given the financial incentives, expectancy bias is a concern. Please acknowledge this limitation explicitly.
Appropriate use of statistics and treatment of uncertainties:
ANCOVA was used as prespecified and effect sizes are reported. However:

The results section is somewhat superficial. Please provide absolute group means and SDs at baseline and follow-up for all outcomes in the main text, not only adjusted differences.
Report effect sizes with 95% CIs for adjusted mean differences, standardized mean differen-ces, and proportions achieving reliable or clinically meaningful change.
Present all effect sizes clearly in tables for visibility.
Comment on the discrepancy between the anticipated effect size (d=0.4) used for power cal-culation and the achieved effects.
The cisgender-specific effects on DERS-8 and GAD-7 were not pre-registered. Given multi-ple outcomes and moderators tested, apply a principled multiplicity strategy to assess robust-ness.
Provide sensitivity analyses to address possible bias from faster engagement decline in the intervention arm.
Report AEs/SAEs by arm to strengthen the safety assessment.
Conclusions: robustness, validity, reliability:
The conclusion that Purrble is "effective" is overstated given design constraints. The lack of an active or placebo comparator and reliance on self-report measures mean expectancy and engagement could account for some of the effects. Results should be framed as preliminary and provisional, requiring replication with stronger controls. The 13-week follow-up limits infe-rences about durability.
Discussion:
• The conclusion that the intervention is effective should be tempered. The short follow-up (13 weeks) limits inference about durability.
• Situate the observed effect size relative to other interventions targeting emotion regu-lation.
• Reflect on scalability: what is the cost of the intervention relative to the achieved ef-fect and its likely durability?
• Include sensitivity analyses addressing differential engagement between arms.
Clarity and context
• The abstract contains a typo ("Purbble" → "Purrble") and should be revised for precision.
• The introduction is somewhat superficial and should more clearly position this intervention within existing literature.
• Abbreviations (e.g. TGD) should be spelled out on first use.
• The power calculation needs clarification: reconcile the protocol's one-sided t-test with the ANCOVA analysis actually performed.
• Discussion should temper claims of effectiveness and reflect on durability, robustness, and generalizability.
• State whether concomitant treatments or therapies were tracked and balanced across arms.
• Acknowledgement: revise wording ("We would like to thank Dr Emma Nielsen for their comments…").
References
The manuscript cites relevant prior work but should include references to comparab-le digital/self-help interventions for emotion regulation and self-harm. This will help contextualize the originality and situate the study within broader intervention re-search.

Reviewer #2

(Remarks to the Author)
This manuscript reports on a randomized controlled trial (RCT) evaluating the effect of the Purrble socially assistive robot on emotion regulation and mental health outcomes among LGBTQ+ youth at risk of self-harm. The trial enrolled a final sample of 153 participants, with 76 randomized to the Purrble intervention and 77 to the waitlist control group. Attrition was low overall, with 7 participants (9.2%) lost to follow-up in the Purrble group and 5 participants (6.5%) in the waitlist group. Statistical tests indicated that attrition rates did not differ significantly between groups, nor by gender identity. The study is interesting and the findings contain some provocative and unexpected result. However, I have several methodological and interpretive concerns that dampen my excitement for the manuscript:

1. The protocol was made available through an article published in BMJ Open. According to the article, the primary analysis was to use a one-sided t-test of mean changes in emotion dysregulation, with linear mixed-effects models reserved for exploratory analyses. In the submitted manuscript, regression models are presented as primary analysis and mixed-effects models are exploratory analyses. While I agree that regression and - especially - mixed-effects modeling represent a more rigorous and appropriate approach than a one-sided t-test, the discrepancy between the pre-specified protocol and the reported analytic strategy raises some concerns. The authors should explicitly justify these deviations, and provide either the original protocol submitted to IRB or a transparent account of all changes to the analysis plan.

2. The manuscript does not clearly describe whether main effects were included alongside the reported interaction terms. The provided Tables are quite difficult to read, with respect to typical reporting uses. An interaction term is only interpretable relative to the inclusion of the corresponding main effects; otherwise, the model implicitly assumes that the predictors have no effect when the other variable is set to zero. Clarification is needed on whether the regression and linear mixed-effects models included the appropriate main effects and how these were coded.

3. Line 133 and Table 2: The reporting is ambiguous. If p-values are presented, these are inferential results and not purely descriptive statistics. It should be stated clearly what hypothesis is being tested and what the reported p-values represent.

4. Lines 148–149 ("approached significance"): This phrase is misleading and should be avoided. Results that are not statistically significant should be reported as such. For instance, the difference in baseline loneliness between completers and those lost to follow-up ($d = –0.75$, 95% CI [–1.60, 0.09], $p = .079$) can be described as relatively large in magnitude but not statistically significant

5. l. 163–165. The model under discussion can be written as:

$$
Y = \beta_0 + \beta_1 ,\text{Condition} + \beta_2 ,\text{Gender} + \beta_3 (\text{Condition}\times\text{Gender}) + \varepsilon,
$$

where Condition = 0 (waitlist), 1 (Purrble), and Gender = 0 (cisgender), 1 (TGD). Under this coding:
β_1 = the treatment effect for cisgender participants (since Gender = 0),

β_2 = the difference between TGD and cisgender participants in the waitlist group (main effect of gender),

β_3 = the difference in treatment effect for TGD compared to cisgender.

Thus, the treatment effect for cisgender youth is given by β_1, while for TGD youth it is $\beta_1 + \beta_3$. The statement that "condition was not a significant predictor for TGD participants" correctly refers to this combined effect, but it does not address the gender main effect β_2. Moreover, the text risks suggesting that cisgender participants had no post-test difficulties, when the correct interpretation is that they had fewer difficulties relative to controls, conditional on baseline. I recommend clarifying that cisgender participants improved compared to waitlist, while the effect was not significant among TGD participants, and that baseline gender differences are captured separately by β_2. As mentioned before, the tables should probably be revised to provide a non-ambiguous result.

6. l. 179–181. The same interpretative logic applies. The significant reduction in anxiety among cisgender youth reflects β_1, while the non-significant effect among TGD participants reflects $\beta_1 + \beta_3$. This should not be conflated with the main effect of gender (β_2). Please revise the wording accordingly.

7. l. 195–198. Again, the condition effect for cisgender is given by β_1, and for TGD by $\beta_1 + \beta_3$. If the interaction was not significant for depression, this should be stated explicitly, and the interpretation revised to avoid conflating the non-significant condition effect in TGD participants with the absence of gender differences overall.

8. The manuscript acknowledges that the use of a waitlist control is a limitation, and I agree this is an important point. However, it is not clear why a waitlist design was chosen instead of alternatives such as treatment-as-usual, an active comparator, or a placebo control (e.g., a neutral interactive toy).

9. Related to the point above, since the intervention is tangible and novel (a robot that responds interactively), it may have nonspecific psychological effects (attention, novelty, comfort) that are not controlled for. A neutral interactive toy or "sham" device would help disentangle these effects.

10. The intervention requires active use of Purrble, but engagement levels vary. Participants who are more motivated or have stronger support systems may use the device more and also have better outcomes, independent of the device itself. Without a measure of adherence or engagement in the main analyses, it is hard to separate the effect of Purrble from the effect of engagement..

Reviewer #3

(Remarks to the Author)
A. This study reports on findings from an RCT of the Purrble intervention, designed to improve emotion regulation and transdiagnostic risk. The findings indicate support for Purrble in cisgender sexuality diverse youth in terms of improved emotion regulation and reduce depression and anxiety.

B. This study represents a novel and innovative contribution to the literature and a potential scalable intervention to support emotional regulation in young people. While there are emerging digital and AI interventions for this population, to my knowledge there are no other socially assistive robots being evaluated or freely available for this population.

C. The study utilises rigorous methodology and reporting, with strong attention to CONSORT guidelines and justification for deviations. My main concern is around the validity of measures for LGBTQA+ young people, the role that LGBTQA+ youth played in the context of Sprouting Minds, whether the ASIST training was adapted for LGBTQA+ youth and if any adaptations were made for the safety plans. In short, while the intervention was applied to LGBTQA+ young people, I can't see much evidence of tailoring the study to the population, or a rationale as to why this wasn't needed.

D. To my knowledge, analyses were robust and appropriate, with justification clearly outlined.

E. While the conclusions are appropriate, I would caution the authors from stating that Purrble is effective for LGBTQA+ young people if there is limited evidence to support its efficacy with trans and gender diverse youth - perhaps it would be more appropriate to use the term cisgender sexuality diverse young people?

F. I understand that additional information is provided in the supplementary materials, however, I would like to see a more

detailed explanation of the reasoning for why trans and gender diverse young people were not responsive to Purrble. It also makes sense to suggest this as an area for further exploration in future research. Further, what was the rationale for using a Cohen's d of 0.4 in your power analysis?

G. Appropriate referencing

H. The manuscript was very well written with a clear rationale for the study and justification for methodological and analytic decisions. The abstract was clear and accurate (though reference to Purrble supporting LGBTQA+ youth in general may need to be toned down to reflect benefits to the cisgender cohort specifically).

Version 2:

Reviewer comments:

Reviewer #1

(Remarks to the Author)
The authors have addressed my concerns in the manuscript. I have no further comments.

(Remarks on code availability)

Reviewer #2

(Remarks to the Author)

(Remarks on code availability)

Reviewer #3

(Remarks to the Author)
Thank you to the authors for your responses to my comments. Overall, I found the responses to be satisfactory, however, I have a couple of minor points that authors could address for improved clarity before final acceptance:
- Re: comment C - the revised text describing the role of the advisory group doesn't fully specify the role of LGBTQA+ lived experience within the group. You note that only 1 member of the advisory group identified as LGBTQA+ - this should be explicit (or at least noted as a % of the group) and could be mentioned in the limitations section. In particular, trans membership of an advisory group would be critical to future research in this space.
- Re: comment F - the revised text on page 44 outlining the rationale for effect size should include citations to the 'previous intervention research with and without Purrble'.

(Remarks on code availability)
N/A

Open Access This Peer Review File is licensed under a Creative Commons Attribution 4.0 International License, which permits use, sharing, adaptation, distribution and reproduction in any medium or format, as long as you give appropriate credit to the original author(s) and the source, provide a link to the Creative Commons license, and indicate if changes were made.
In cases where reviewers are anonymous, credit should be given to 'Anonymous Referee' and the source.
The images or other third party material in this Peer Review File are included in the article's Creative Commons license, unless indicated otherwise in a credit line to the material. If material is not included in the article's Creative Commons license and your intended use is not permitted by statutory regulation or exceeds the permitted use, you will need to obtain permission directly from the copyright holder.
To view a copy of this license, visit https://creativecommons.org/licenses/by/4.0/

Response to Referees

We thank the editor and reviewers for their careful consideration of our manuscript, NMED-A143742B: "Effects of a Socially Assistive Robot on Emotion Regulation and Mental Health in LGBTQ+ Young People at Risk of Self-Harm: A Randomised Controlled Trial", and for these constructive comments. We have revised the manuscript accordingly and address each point in turn below. Editorial/reviewer comments are reproduced in italics, followed by our responses and indications of where changes have been made in the manuscript, as applicable.

Editor

Editorial Comment: *Abstract should be ~200 words and for trials should adhere to the CONSORT framework. You must state if the primary outcome was or was not met and provide the effect size and relevant uncertainty estimate. The conclusion of the study must focus only on the primary outcome and safety/tolerability. You must report either all or none of the secondary outcomes, given the space limitation, I would suggest removing all mention of secondary outcomes from the abstract.*

- We have revised the abstract to align with the CONSORT framework. Specifically, we now explicitly state that the primary outcome was met, report the effect, its 95% confidence interval, and effect size, and comment on safety/tolerability. In line with your guidance and the word limit, we have removed all references to secondary outcomes (e.g., depression, anxiety) from the abstract.

Editorial Comment: *Please include the trial registration number at the end of the Abstract.*

- We have included the trial registration number at the end of the abstract: “ClinicalTrials.gov: NCT06025942.”

Editorial Comment: *The Introduction should be written for a broad, non-specialist medical reader and provide sufficient context for the work.*

- We have made targeted revisions to the Introduction to provide additional context on socially assistive robots for a broad, non-specialist readership.
- **Associated Text (page 6):** One emerging and promising approach involves the use of socially assistive robots (SARs), which provide support to a user through social interactions. SARs have been evaluated in various contexts, including education, family, and healthcare settings with children[17-20], and have shown encouraging outcomes in enhancing motivation, supporting skill development, and improving predictors of poor mental health, such as loneliness and stress[17-22].

Editorial Comment: *Please provide details of your cohort in the first paragraph of the Results. This includes number of individuals screened for enrolment, as well as exact dates of first and last patient enrolment. The CONSORT patient disposition diagram and the baseline*

characteristics must be included as main non-text items (for which there is a strict limit of 6 tables and/or figures).

- Thank you for this clarification. We have added a Participant Disposition subsection at the beginning of the Results, including the number of individuals screened and the exact dates of first and last participant enrolment. We have intentionally avoided the term "patient" to minimise stigma and instead use "participant," but we are happy to adjust this terminology if preferred. We have also reduced the number of main tables and figures to six, with the CONSORT diagram and baseline characteristics now included among them.
- **Corresponding Text/Sections:**
  - See Participant Disposition, beginning on page 8
  - See CONSORT Diagram, page 27
  - See Table 1 for participant characteristics, page 29
  - See Table 2 for baseline characteristics, page 30

Editorial Comment: *The Results should be structured as followed:*
- Patient disposition
- Primary outcome(s)
- Secondary outcomes
- Safety
- Exploratory outcomes
- Sensitivity analyses
- Post-hoc analyses

- We have substantially reorganised the Results section to follow this structure. All analyses are now presented in the order specified.

Editorial Comment: *Please remove any subheadings from the Discussion.*

- All subheadings have been removed from the Discussion section.

Editorial Comment: *Please ensure all results are presented in the Results section, no new data should be introduced in the Discussion.*

- We have reviewed the Discussion and confirm that no new data are introduced; all results are presented in the Results section.

Editorial Comment: *You must include explicit paragraphs of study limitations in the Discussion.*

- In line with the previous version of the manuscript, the Discussion already contains explicit paragraphs detailing study limitations. We have reviewed and retained these to ensure the limitations are clearly delineated.

Editorial Comment: *The overarching conclusion of the study must be based only on the primary outcome and safety data.*

- We have revised the abstract and the overarching conclusion in the Discussion to focus exclusively on the primary outcome and safety/tolerability data.

Editorial Comment: *The Methods should include a full description of the inclusion and exclusion criteria, as well as study procedures and statistical analyses (including a power calculation).*

- We have expanded the description of inclusion criteria and clarified that there were no exclusions based on concurrent mental health services, improving interpretability.
- We have expanded the power analysis section to report both the a priori power calculation (used for sample size estimation) and the achieved power for our primary ANCOVA analysis.
- **Associated Text**
    - **(page 34):** It was not necessary for participants to indicate whether they were receiving any additional treatments or therapies for their mental health difficulties.
    - **(page 44):** *begins with…* Because our preregistered analytic plan specified t-tests and we subsequently adopted an ANCOVA framework, we examined the effective power of the final sample under the ANCOVA specification.

Editorial Comment: *Please upload the protocol and SAP with the revision materials, so that reviewers and editors have access to them.*

- We have uploaded the trial protocol, including the statistical analysis plan (SAP), with the revision materials.

Editorial Comment: *You must ensure that contributions from all individuals in the author list are available in the Author Contributions statement.*

- We confirm that the Author Contributions statement has been updated to include contributions from all authors listed on the manuscript.

Editorial Comment: *Please move all funding sources to the Acknowledgements, including a statement on the role of the funder.*

- All funding sources have been moved to the Acknowledgements section, and we now explicitly describe the role of the funder.

Editorial Comment: *Please ensure that all potential competing interests are detailed for all authors. For any authors with no competing interests, this must also be stated.*

- We have reviewed and updated the Competing Interests statement to ensure that all potential competing interests are reported and that authors without competing interests are explicitly identified as having none.

Editorial Comment: *Please see our guidelines for the Data and Code Availability Statements. "Available on request" is not acceptable, you must provide details of any restrictions to data and code availability*

- Thank you for this important comment. We have revised the Data and Code Availability Statements to clarify the specific conditions under which data can be shared. In particular, we now distinguish between datasets that can be shared without restriction (e.g., study data with all demographic variables removed) and datasets that contain demographic information. Because Purrble is a physical, visually recognisable device and participants identified as gender and/or sexual minorities and reported on highly sensitive outcomes (self-harm and mental health), there is a heightened risk of re-identification when demographic variables (e.g., gender identity, sexuality, age, race) are included. For this reason, de-identified datasets without demographic information will be made available upon reasonable request, whereas access to datasets including demographic variables will require a data use agreement outlining appropriate safeguards. Analysis code will be made available to any requester without restriction.
- **Associated Text (page 51):** *begins with…* Because the intervention involved a physical, visually recognisable device (Purrble),

Editorial Comment: *The article file must only contain these items in this order:*
• Title
• Author List and affiliations
• Abstract
• Introduction
• Results (with Subheadings)
• Discussion
• Acknowledgements
• Author Contributions
• Competing Interests Statement
• References (for main text only)
• Figure legends (for main text only)
• Tables (note: tables should be pasted into Word files as editable tables, not as images)
• Methods
• Data Availability Statement
• Code Availability Statement
• Methods-only References

- We have reordered the article file to match the sequence specified. The only addition is an Extended Data section, which we have placed after the Methods-only references, consistent

with the online formatting guidance. This section allows us to include tables requested by reviewers and to present exploratory analyses without exceeding the main-text limit on tables and figures.

Reviewer 1

In general, the study design (intervention vs. waitlist, unblinded self-report outcomes) limits the strength of the conclusions. Participants knew allocation, the waitlist would receive Purrble after week 13, and all participants could keep devices. Together with £5 per survey, this may have amplified expectancy and engagement differences. Waitlist designs are vulnerable to inflated effects on self-report outcomes. Claims should therefore be tempered and framed as provisional until replicated with an active or placebo control.

- Thank you for this critical comment. We agree that the waitlist design introduces important methodological limitations, and we have acknowledged this explicitly in the revised Limitations section. In revising the manuscript in light of this comment, we also realised that our original framing did not adequately describe the clinically active components of the intervention other than allocation to Purrble. Specifically, we had only briefly mentioned in the procedures section that the safety debriefing session included safety planning, without clearly conveying that all participants (in both arms) received a structured safety-planning intervention that is broadly consistent with treatment-as-usual for self-harm in many care settings.

  We have therefore revised the manuscript to more accurately reflect the nature of the conditions and to temper our conclusions accordingly. First, we have renamed the trial arms throughout from “Purrble vs. waitlist” to “Purrble plus safety planning vs. safety planning only,” to foreground the active safety-planning component. Second, we have added text to the Introduction to describe safety planning and its clinical relevance, and we have substantially expanded the Methods section to provide a clearer description of what occurred in the safety-planning/debrief session and how safety planning was used as part of our safeguarding procedures.

  In addition, we now explicitly describe how the trial design was co-developed with our youth advisory group, Sprouting Minds. Advisors strongly advocated for a waitlist control as the most ecologically valid comparison for LGBTQ+ young people’s typical experience of waiting for mental health services. On this basis, and because participants in the comparison arm received only our standardised safeguarding procedure (safety planning), we have clarified that this condition is best conceptualised as “safety planning only” and can be conceptualized as a treatment-as-usual comparator within the trial.

  Notwithstanding these clarifications, we agree that the “waiting for Purrble” element still introduces methodological challenges, including expectancy and engagement differences between groups. In the revised Limitations section, we now discuss this explicitly, highlight the vulnerability of self-report outcomes in this context, and further emphasize

that conclusions should be considered provisional until replicated in future trials using more robust comparison conditions (e.g., ongoing active control or placebo control).

- **Associated Text in Introduction (page 7):** In the current pre-registered RCT (ClinicalTrials.gov: NCT06025942), we evaluated the efficacy of safety planning and access to Purrble (Purrble + SP) in improving emotion regulation and reducing mental health symptom severity in a 13-week trial with LGBTQ+ young people experiencing thoughts of self-harm, compared with safety planning alone (SP-only). Safety planning, a brief collaborative intervention involving identifying warning signs, coping strategies, and sources of support[29], is recommended by multiple guiding bodies as standard ("treatment as usual") care for self-harm and suicidal presentations across practice settings [30, 31]. For this reason, we included safety planning as a standard safeguarding procedure across conditions.
- **Associated Text in Discussion (page 18):** Participants were aware of their group assignment and that the safety planning–only group would later receive Purrble[46], creating potential expectancy and engagement differences that may inflate self-reported improvements and limit causal inference. Moreover, there was no sham or placebo device to isolate Purrble-specific mechanisms (e.g., socially assistive feedback) from non-specific factors such as novelty or having any comforting object. Future trials would benefit from more active control conditions, such as clearly documented treatment-as-usual comparators[47] or, where feasible, attention-matched or placebo device controls that more stringently test the added value of the socially assistive components of Purrble.
- **Associated Text in Procedures (page 35):** Advisors from Sprouting Minds critically reviewed the trial's methodology, focusing particularly on participant burden and the frequency and duration of survey assessments. The final design — weekly surveys lasting approximately 5-10 minutes over 13 weeks, with a reimbursement of £5 per survey — was deemed acceptable and fair by advisors. Additionally, advisors informed the selection of the control group, strongly advocating for a waitlist control condition. They noted that while allocation to a waitlist could be initially discouraging for participants, it accurately reflected real-world experiences associated with waiting periods for mental health services. Thus, any dropout observed in this group would realistically represent typical treatment disengagement. As participants in this condition received only our standardised safeguarding procedure (i.e. safety planning), it was classified as treatment-as-usual for the purposes of the trial.
- **Associated Text:** See ***Compulsory Safety Planning*** section (page 36-37).

If expectancy measures were collected, these should be adjusted for or, if not, the absence should be discussed.

- Expectancy measures were not collected in this study. Because Purrble was not described or positioned as a therapeutic program or skills-based intervention, it was not appropriate to administer standard expectancy measures (e.g., Credibility/Expectancy Questionnaire; Devilly & Borkovec, 2000), which explicitly reference “therapy,” “treatment,” and/or “program.” Participants were unfamiliar with Purrble at enrollment and were encouraged simply to explore the device rather than expect specific benefits.
- We have specifically addressed this point in the measures section within the Methods:
    - **Associated Text (page 39):** Expectancy measures were not collected, as Purrble was not introduced or described as a therapeutic program or skills-based intervention, and standard expectancy instruments (e.g., Credibility/Expectancy Questionnaire[62]) would not have been appropriate in this context. Participants were unfamiliar with Purrble at enrolment and were encouraged simply to explore the device and were not primed to expect specific benefits.

Originality and significance

The intervention is novel in targeting emotion regulation through a socially assistive robot in a high-risk LGBTQ+ population. This is an important and underserved group. To my knowledge, few if any RCTs have tested similar interventions in this setting. The work has the potential to be clinically meaningful if replicated with more rigorous controls.

- We thank the reviewer for highlighting the study's originality and significance. The potential to support emotion regulation in this high-risk and underserved LGBTQ+ population using a novel approach was the primary motivator for this work. We agree with the reviewer's assessment that these findings, while preliminary, are clinically meaningful and provide a crucial foundation for the more rigorous, controlled trials that are necessary to follow.
- **Associated Text (page 19):** Taken together, these findings position Purrble as a feasible, scalable adjunct to usual care or waitlist periods for cisgender sexual-minority youth, while underscoring the need for targeted co-design and evaluation to meet the distinct needs of TGD youth. In order to enhance generalisability and support real-world implementation, future work should employ placebo control conditions, test mechanisms of change, extend follow-up, and evaluate integration alongside evidence-based therapies and service pathways.

The introduction is somewhat superficial. Please situate the work more clearly within prior literature: have other innovative interventions been trialed in this population, and how does this study extend or deviate from existing approaches?

- We fully agree that greater context would strengthen the introduction. To address this, we have added the following text to situate our work within prior literature:

- **Associated Text (page 5):** Digital mental health interventions are widely proposed as a scalable solution to address these gaps in support and, among LGBTQ+ youth, such interventions are generally rated as feasible and acceptable[14]. A recent review suggests that digital approaches embedded in service settings (e.g., Zoom-based therapy with a provider) and/or providing explicit psychoeducation and skill development (e.g., self-guided adaptations of evidence-based therapies) show the most promising evidence for reducing psychopathology in LGBTQ+ youth, though none have evaluated self-harm as an outcome[15]. Digital supports that lack formal, structured components remain underdeveloped and, when evaluated in this population, tend to show weaker and highly variable effects[15]. Outside of the context of tightly controlled trials, engagement and retention with such digital interventions in real-world settings are often insufficient to derive clinical benefit[16], reflecting systemic barriers to access, privacy, and sustained use. This mismatch between the promise of these interventions and their low real-world uptake underscores the need for approaches that promote clinically meaningful skills and are intrinsically easy to weave into real-world routines.

Data & methodology

Ensure all abbreviations are introduced (e.g. "TGD" appears in the Results without definition).

- Thank you for noting this omission. The abbreviation has been defined at first use to read "transgender or gender diverse [TGD]." (page 8)

Please justify the age range (16–25).

- Thank you for highlighting this. We have clarified the rationale for the age range in the manuscript. Briefly, we selected 16–25 years for ethical, developmental, and clinical reasons. Ethically, 16 corresponds to the UK age of consent, enabling young people to provide informed consent to participate without requiring parental involvement. Developmentally, 25 aligns with commonly used definitions of youth and emerging adulthood, and with the National Youth Agency's definition of youth (11–25 years). Clinically, this range captures a developmental window in which self-harm typically emerges and peaks, making it a critical period for targeted intervention.

- **Associated Text (page 34):** Eligible participants were individuals aged 16–25 years (inclusive). The lower bound was chosen to begin at the UK age of consent, and the upper bound to align with the National Youth Agency's definition of youth (11–25 years) [52]. This range captures the developmental period when self-harm is likely to reach its peak[53,54].

Clarify whether a cut-off for self-harm ideation severity was required for inclusion, as this affects generalizability.

- Thank you for this helpful comment. We have clarified the inclusion criteria in the manuscript to specify that participants were required to endorse any level of self-harm ideation or behavior on the screening measure in the preceding month to be eligible. There was no upper limit or severity cut-off applied.
- **Associated Text (page 34): ...** and reporting any experiences of self-harm ideation or behaviour within the previous month (i.e., any response greater than "none" on any questions on the screener), with no upper severity limit.

Report the response rate to weekly surveys over time. Declining engagement is common in mental health populations and raises risk of selective reporting.

- Thank you for this important point. We have now included a table summarizing weekly participation rates by condition across the study period in the Extended Data section.
- **Associated Text (page 57):** See Extended Data Table 1.

Provide details on the qualitative process analyses: how many participants were included, what were their characteristics, and do they reflect the overall sample?

- We appreciate this helpful comment. The reviewer rightly points out that a proper interpretation of the qualitative process analyses would require significant additional detail on the sample and methodology.
  Upon reflection, we believe that these exploratory findings deserve a more in-depth analysis than is possible within the scope of this primarily quantitative paper. To avoid a superficial treatment that could lead to misinterpretation, we have elected to withdraw the qualitative process analysis and any associated conclusions from the current manuscript. This decision allows us to present those findings with the appropriate methodological context in a separate publication, while keeping the present paper focused on the quantitative trial outcomes.

Confirm whether a statistical analysis plan was published in addition to the protocol.

- We can confirm that no separate statistical analysis plan was published beyond the trial protocol.

Randomisation requires fuller reporting: how was the sequence generated, was blocking or stratification used (beyond gender identity), who had access to the sequence, and what safeguards prevented foreknowledge or manipulation? Was allocation performed after consent and baseline assessments?

- We appreciate this important comment and have expanded the manuscript to clearly describe the randomisation process in full detail. The revised text now specifies how the randomisation sequence was generated, the use of stratification (by gender identity), who

had access to the allocation sequence, the safeguards in place to prevent foreknowledge or manipulation, and that randomisation/allocation occurred only after consent and baseline data collection.

- **Associated Text (page 8):** Randomisation was conducted after consent and baseline data collection (Week 3) by the study coordinator, who was the only person with access to the randomisation process. Participants were grouped into blocks based on similar enrolment timeframes. Within each block, participants were individually randomised in a 1:1 ratio (stratified by gender identity, cisgender vs. transgender or gender diverse [TGD], to ensure balance between conditions) to either the Purrble and safety planning condition (Purrble + SP) or the safety planning + waitlist (SP-only) condition, using an online randomisation generator.

Outcomes appear to rely exclusively on self-report. Without blinded observer-rated measures, and given the financial incentives, expectancy bias is a concern. Please acknowledge this limitation explicitly.

- Thank you for this helpful observation. We agree that reliance on self-report measures and the use of financial incentives may have introduced expectancy bias. This has been explicitly acknowledged in the manuscript in the limitations section as follows:
- **Associated Text (page 18):** Second, participants were aware of their group assignment and that the safety planning–only group would later receive Purrble[46], creating potential expectancy and engagement differences that may inflate self-reported improvements and limit causal inference.

Appropriate use of statistics and treatment of uncertainties:

ANCOVA was used as prespecified and effect sizes are reported. However: The results section is somewhat superficial. Please provide absolute group means and SDs at baseline and follow-up for all outcomes in the main text, not only adjusted differences.

- Thank you for this helpful suggestion. We have added a new table in presenting baseline and follow-up means and standard deviations for all continuous outcomes by condition which we refer to at the beginning of the results section (page 30).

Report effect sizes with 95% CIs for adjusted mean differences, standardized mean differences, and proportions achieving reliable or clinically meaningful change.

- Thank you for this constructive recommendation to enhance our reporting of effect sizes. In response, we have substantially expanded our results to provide a more comprehensive picture of the intervention's effects.
  - First, as requested, we now report the adjusted mean differences from our ANCOVA models, along with their corresponding 95% confidence intervals.

  - Second, we have also conducted and added a reliable change analysis (see Table 2 on Page 30). In the manuscript, we now report the proportion of participants in each group who achieved reliable improvement, accompanied by odds ratios (and their 95% CIs) to statistically test for differences in the likelihood of achieving this change between groups.
  - Third, we have added 95% CIs for all our effect size η_p^2 values and explicitly quantify their magnitude (e.g., "a medium effect") in the text.
  - Finally, regarding the request for standardized mean differences (e.g., Cohen's *d*), after careful reflection, we have opted to retain partial eta squared (η_p^2) as the primary effect size for our ANCOVA models. Our rationale is that η_p^2 is the most appropriate and widely accepted effect size for ANCOVA, as it directly quantifies the proportion of variance in the outcome that is uniquely attributable to the intervention group, after partialling out the variance explained by the covariate(s). A standard Cohen's *d* based on unadjusted means would not be appropriate, as it would fail to account for the baseline differences our model was designed to control for. While an adjusted SMD could be computed, η_p^2 is a more direct and standard measure of the effect's magnitude *within the context of this specific model*.
- We believe this combination of adjusted mean differences, reliable change proportions, and η_p^2 (all with 95% CIs) provides a robust and statistically appropriate account of the findings. However, we are open to including an adjusted standardized mean difference in a future revision if it is requested.
- **Associated Text: (page 48):** For all main effects analyses except self-harm, we examined individual-level change using reliable change indices (RCIs) for emotion regulation difficulties (DERS-8), anxiety (GAD-7), and depressive symptoms (PHQ-9)[94]. For each scale, we used the baseline standard deviation and its internal consistency (Cronbach's α) as the reliability estimate to derive an RCI score for each participant, and classified outcomes as reliable improvement, reliable deterioration, or no reliable change based on the ±1.96 criterion. We then compared conditions using odds ratios with 95% confidence intervals, estimating (a) the odds of reliable improvement versus all other outcomes and (b) the odds of reliable deterioration versus all other outcomes for Purrble + safety planning relative to safety planning alone.

Present all effect sizes clearly in tables for visibility.

- We have revised the tables to present all effect sizes along with their corresponding 95% confidence intervals to enhance clarity and visibility.

Comment on the discrepancy between the anticipated effect size (d=0.4) used for power calculation and the achieved effects.

- For our primary outcome (DERS-8), the ANCOVA yielded a partial η^2 **= .09**. This is conventionally considered a **medium-to-large effect size** and is therefore consistent in magnitude with the 'medium' effect we anticipated in our *a priori* power analysis. To further address this comment, we calculated *Cohen's d* for the emotion regulation outcome, which was 0.54 [95% CI = 0.20, 0.88]. Thus, the achieved effect is closely aligned with the effect size used for the power calculation. We have added text to explicitly address this alignment in the discussion section.
- Additionally, given the shift from t-test to ANCOVA, we conducted a new power calculation to identify our achieved power. Analyses revealed that with our sample size, a-priori effect size estimate, and three covariates, we achieved 91% power to detect an effect.
- **Associated Text**
    - **Discussion (page 16):** The emotion regulation effect size (partial η^2 = .09) was medium-to-large, mirroring meta-analytic effects for skills-based interventions (d ≈ .36)[34] and aligning with our a-priori benchmark (d = .40).
    - **Methods (page 44):** Because our preregistered analytic plan specified t-tests and we subsequently adopted an ANCOVA framework, we examined the effective power of the final sample under the ANCOVA specification. Using Shieh's exact method for ANCOVA (ANCOVA_analytic in the Superpower package, R) with the observed post-intervention DERS means in the waitlist and Purrble conditions (M = 25.26 and 28.61, pooled SD = 7.34), three covariates (baseline DERS, age, gender identity), and the empirically estimated covariate R^2 = 0.38, the achieved sample size yielded an estimated power of 91.7% to detect the observed emotion regulation effect at α = .05.

The cisgender-specific effects on DERS-8 and GAD-7 were not pre-registered. Given multiple outcomes and moderators tested, apply a principled multiplicity strategy to assess robust-ness.

- Thank you for this helpful suggestion. We conducted robustness checks using the Benjamini–Hochberg (BH) correction to control for false discovery rate across both our primary outcome analyses and the moderation analyses. For the three continuous outcomes, applying the BH adjustment did not change any conclusions (adjusted *p*s = .003, .044, .000). For the moderation analyses, all three *p*-values were no longer significant after correction (adjusted *p*s = .057, .057, .076). We have incorporated this information into the *Results* and discussed its implications in the *Discussion* section. Specifically, we note that this correction calls into question the stability of the moderation effects. However, we also emphasize that these analyses were underpowered and that all moderation effects trended consistently in the same direction. Simple slopes analyses further supported the presence of differential effects by gender identity. While replication

in a larger sample is warranted, these findings suggest that treating LGBTQ+ participants as a homogenous group may obscure meaningful subgroup differences.

- **Associated Text:**
  - **Results (page 14-15):** To evaluate robustness to multiple testing, we applied the Benjamini–Hochberg correction separately within each family of analyses. Specifically, we corrected for the three primary continuous outcomes (DERS-8, GAD-7, PHQ-9) and, in a separate step, for the three moderation models examining condition × gender identity interactions. The moderation analyses were exploratory and not pre-registered. For the continuous outcomes, significance levels were unchanged after correction (adjusted *ps* = .003, .044, .000). For the moderation analyses, all effects were no longer statistically significant after correction (adjusted *ps* = .057, .057, .076).
  - **Discussion (page 16-17):** While the Benjamini–Hochberg corrections provided a more conservative test of significance, they also indicated that the moderation effects were not robust to multiplicity adjustment. These moderation analyses were exploratory and not pre-registered, and the study was underpowered to detect moderated effects with adequate precision. Nonetheless, the consistency and directionality of these findings, supported by simple slopes analyses, suggest that subgroup differences by gender identity may be meaningful and merit replication in larger, adequately powered trials.

Include sensitivity analyses addressing differential engagement between arms.

- Thank you for this helpful suggestion. Although there was only a small absolute difference in attrition between arms (a two-participant difference), we agree that it is important to formally assess whether differential engagement could bias the findings. We have therefore added a set of sensitivity analyses to the Results section.

  First, we examined whether attrition differed by condition or gender identity, and whether attrition was associated with any baseline variables. Chi-square tests indicated that binary attrition (completer vs. non-completer) did not differ significantly by condition, $\chi^2(1) = 0.11$, $p = .75$, or by gender identity, $\chi^2(1) < 0.01$, $p > .99$, and there were no main or interactive effects of attrition status on any baseline variable. This suggests no evidence of systematic differential attrition between arms.

  Second, to address potential differences in engagement with the weekly surveys, we re-estimated all primary outcome models including total number of survey responses as a covariate (i.e., an index of adherence/engagement). Total survey responses did not significantly predict outcomes in any model, and the magnitude, direction, and significance of the primary intervention effects were unchanged. Together, these analyses suggest that the findings are robust to the small observed differences in weekly response between arms.

- **Associated Text (page 15):** To assess the robustness of the findings against the observed differential decline in survey responses, we conducted several sensitivity analyses. Firstly, chi-square tests indicated that binary attrition rates did not differ significantly by condition, $\chi^2(1) = 0.11$, $p = .75$, or by gender identity, $\chi^2(1) < 0.01$, $p > .99$, and no main or interactive effects of attrition status were observed on any baseline variable, indicating no evidence of differential attrition by condition. To further account for variations by condition in adherence, we re-estimated all the main effects models including total number of survey responses as a covariate. This covariate was not a significant predictor in any model, and the significance, magnitude, and direction of the primary effects remained unchanged.

Report AEs/SAEs by arm to strengthen the safety assessment:

- Thank you for this helpful comment. In line with the recommendation, we have expanded the manuscript to report adverse events (Aes) and serious adverse events (SAEs) by trial arm.
- **Associated Text (page 11-12):** Three participants required reactive safeguarding for adverse event during the trial: one from the Purrble + SP condition and two from the SP-only condition… During these safeguarding contacts, participants were asked about their current emotional state and whether they felt at risk of self-harm or a suicide attempt. In all cases, participants attributed their increased distress to life events external to the trial, and no imminent risk was identified. The researcher reviewed each participant's safety plan and updated it to align with their current needs and circumstances. Participants were also advised to contact relevant support services (e.g., GP, Kooth, Samaritans) to help manage their distress. The participant in the Purrble + SP condition reported feeling guilty about not engaging with the device. They were reassured that there is no "right" way to use Purrble, encouraged to switch off the device, and advised to re-engage only if and when they felt comfortable. No serious adverse events occurred.

Conclusions: robustness, validity, reliability:

The conclusion that Purrble is "effective" is overstated given design constraints. The lack of an active or placebo comparator and reliance on self-report measures mean expectancy and engagement could account for some of the effects. Results should be framed as preliminary and provisional, requiring replication with stronger controls. The 13-week follow-up limits inferences about durability.

- Thank you for this thoughtful comment. We agree that conclusions should be framed cautiously given the study's design and methodological constraints. We have revised the

final paragraph of the *Discussion* to temper claims of effectiveness and to emphasize the preliminary nature of the findings and the need for replication. The revised text now reads as follows:

- **Associated Text (page 19):** This pre-registered, stratified RCT provides the first evidence that an in-situ socially assistive robot can improve mental health-relevant outcomes for LGBTQ+ youth with self-harm thoughts. Across 13 weeks, safety planning + Purrble produced meaningful reductions in emotion regulation difficulties (primary outcome; medium-to-large magnitude) and concomitant decreases in depressive and anxiety symptoms compared with safety planning alone, achieved without any therapist contact or explicit skills training. Benefits were concentrated among cisgender participants; exploratory moderation signals suggested diminished or absent effects for TGD youth. Taken together, these findings position Purrble as a feasible, scalable adjunct to usual care or waitlist periods for cisgender sexual-minority youth, while underscoring the need for targeted co-design and evaluation to meet the distinct needs of TGD youth. In order to enhance generalisability and support real-world implementation, future work should employ placebo control conditions, test mechanisms of change, extend follow-up, and evaluate integration alongside evidence-based therapies and service pathways.

Discussion:

The conclusion that the intervention is effective should be tempered. The short follow-up (13 weeks) limits inference about durability.

- Please see our above response (including mention of future directions to extend follow-up).

Situate the observed effect size relative to other interventions targeting emotion regu-lation.

- Thank you for this suggestion. We now situate our observed effect size relative to the broader literature on emotion-regulation interventions. In brief, our primary outcome showed a medium-to-large effect (partial η^2 **= .09**.), which is modestly larger than, but broadly consistent with, meta-analytic estimates for emotion-regulation interventions (average $d \approx 0.36$; “medium” magnitude). We have added text to the Discussion clarifying that our estimate falls within the range typically reported across trials.
- **Associated Text (page 16):** The emotion regulation effect size (partial $\eta^2 = .09$) was medium-to-large, mirroring meta-analytic effects for skills-based interventions ($d \approx .36$)[34] and aligning with our a-priori benchmark ($d = .40$)

Reflect on scalability: what is the cost of the intervention relative to the achieved effect and its likely durability?

- In the revised manuscript, we have expanded our discussion of scalability to (a) compare the approximate per-participant cost of Purrble to typical costs associated with self-harm–related hospital presentations, (b) explicitly note that the intervention involves a one-off device cost rather than ongoing per-session delivery costs, and (c) acknowledge that the durability of effects beyond the 13-week follow-up remains unknown.
- **Associated text**
    - **Discussion (page 16):** From an economic perspective, the wholesale cost of providing Purrble (~£25 plus ~£6 shipping per unit) is a small fraction of the mean general hospital cost of a single self-harm episode in England (~£809)[35]. Taken together, Purrble may offer a distinct implementation pathway that is scalable, low-burden, and easily accessible in order to expand reach and equity.
    - **Limitations (page 18):** Fourth, the lack of a long-term follow-up limits conclusions about the durability of effects.

Provide sensitivity analyses to address possible bias from faster engagement decline in the intervention arm.

- Please see our response to the previous comment requesting these analyses for a thorough response. (See page 14-15)

Clarity and context

The abstract contains a typo ("Purbble" → "Purrble") and should be revised for precision.

- Thank you for catching this error; we have amended this misspelling.

The introduction is somewhat superficial and should more clearly position this intervention within existing literature.

- Please see our response to a similar comment in the "Originality & Significance" section.

Abbreviations (e.g. TGD) should be spelled out on first use.

- Thank you for noting this omission. The abbreviation has been defined at first use to read "transgender or gender diverse (TGD)." (page 08)

The power calculation needs clarification: reconcile the protocol's one-sided t-test with the ANCOVA analysis actually performed.

- Given the shift from t-test to ANCOVA, we conducted a new power analysis to identify our achieved power. Analyses revealed that with our sample size, a-priori effect size estimate, and three covariates, we achieved 91% power to detect an effect.

- **Associated Text (page 44):** Because our preregistered analytic plan specified t-tests and we subsequently adopted an ANCOVA framework, we examined the effective power of the final sample under the ANCOVA specification. Using Shieh's exact method for ANCOVA (ANCOVA_analytic in the Superpower package, R) with the observed post-intervention DERS means in the waitlist and Purrble conditions ($M = 25.26$ and 28.61, pooled $SD = 7.34$), three covariates (baseline DERS, age, gender identity), and the empirically estimated covariate $R^2 = 0.38$, the achieved sample size yielded an estimated power of 91.7% to detect the observed emotion regulation effect at $\alpha = .05$.

Discussion should temper claims of effectiveness and reflect on durability, robustness, and generalizability.

- Thank you for this valuable comment. We have revised the *Discussion* section to more carefully contextualize the findings and temper claims of effectiveness. We have also noted limitations about durability, robustness, and generalizability.
- **Associated Text (page 18):** Results should be interpreted in the context of several limitations. First, our design compared Purrble and safety planning with safety planning alone, with the safety planning–only group offered Purrble after follow-up. Although this approach was selected to ensure equitable access and to reflect advisory group preferences for mimicking the lived experience of receiving a single safety-planning contact with little follow-up, it introduces methodological challenges. Second, participants were aware of their group assignment and that the safety planning–only group would later receive Purrble[46], creating potential expectancy and engagement differences that may inflate self-reported improvements and limit causal inference. Third, there was no sham or placebo device to isolate Purrble-specific mechanisms (e.g., socially assistive feedback) from non-specific factors such as novelty or having any comforting object. Fourth, the lack of a long-term follow-up limits conclusions about the durability of effects.

State whether concomitant treatments or therapies were tracked and balanced across arms.

- Thank you for this comment. We have clarified this in the methods section:
- **Associated Text (page 34):** It was not necessary for participants to indicate whether they were receiving any additional treatments or therapies for their mental health difficulties. This design decision was intentional to reflect real-world conditions in which individuals might use *Purrble* either alongside, or independently of, other supports or interventions.

Acknowledgement: revise wording ("We would like to thank Dr Emma Nielsen for their comments…").

- Thank you for this consideration. We are not certain which aspect of the phrasing was viewed as inappropriate, but are happy to revise the acknowledgement if the reviewer can clarify the specific concern.

References

The manuscript cites relevant prior work but should include references to comparable-le digital/self-help interventions for emotion regulation and self-harm. This will help contextualize the originality and situate the study within broader intervention re-search.

- Thank you for this helpful comment. We have expanded the introduction to more clearly situate our work within the broader context of digital and innovative interventions for LGBTQ+ populations. The revised text outlines the current state of digital interventions, most of which rely on psychoeducation, social support, or skills training, and emphasizes the lack of rigorous evaluation and absence of self-harm outcomes in this literature. We also introduce socially assistive robots (SARs) as an emerging and distinct approach that differs from traditional digital or self-guided programs by focusing on in-the-moment emotional support rather than skills instruction. This addition helps clarify both the novelty of the present study and its conceptual distinction from prior intervention models.
- **Associated Text (pages 6-7):** Digital mental health interventions are widely proposed as a scalable solution to address these gaps in support and, among LGBTQ+ youth, such interventions are generally rated as feasible and acceptable[14]. A recent review suggests that digital approaches embedded in service settings (e.g., Zoom-based therapy with a provider) and/or providing explicit psychoeducation and skill development (e.g., self-guided adaptations of evidence-based therapies) show the most promising evidence for reducing psychopathology in LGBTQ+ youth, though none have evaluated self-harm as an outcome[15]. Digital supports that lack formal, structured components remain underdeveloped and, when evaluated in this population, tend to show weaker and highly variable effects[15]. Outside of the context of tightly controlled trials, engagement and retention with such digital interventions in real-world settings are often insufficient to derive clinical benefit[16], reflecting systemic barriers to access, privacy, and sustained use. This mismatch between the promise of these interventions and their low real-world uptake underscores the need for approaches that promote clinically meaningful skills and are intrinsically easy to weave into real-world routines.
  One emerging and promising approach involves the use of socially assistive robots (SARs), which provide support to a user through social interactions. SARs have been evaluated in various contexts, including education, family, and healthcare settings with children[17-20], and have shown encouraging outcomes in enhancing motivation, supporting skill development, and improving predictors of poor mental health, such as loneliness and stress[17-22]. One notable example is Purrble, a plush SAR, developed through co-design with youth[23]. The primary target of this intervention is emotion regulation, a known

transdiagnostic risk factor for poor mental health outcomes[24], including self-harm[25,26]. Unlike traditional emotion regulation interventions that rely on in-session, therapist-guided skill-building and psychoeducation[27], Purrble inherently facilitates immediate emotion regulation through intuitive interaction alone, requiring no explicit instruction or prior orientation[17,23]. Purrble simulates a heartbeat that responds to touch, transitioning from frantic vibrations to a calming "purr" as it is soothed by the user (see the intervention section in Methods for a more thorough description). This novel, real-time intervention offers private, ongoing, and consistently accessible support which could address the aforementioned unmet needs. Indeed, LGBTQ+ youth at risk of self-harm have indicated that engaging with Purrble is both feasible and acceptable, with recent pilot work demonstrating its potential to interrupt self-harmful thoughts and behaviours by promoting real-time use/implementation of emotion regulation strategies[28]. However, to date, no randomised controlled trials (RCTs) evaluating the efficacy of the Purrble intervention have been conducted.

Reviewer #2 (Remarks to the Author):

1. The protocol was made available through an article published in BMJ Open. According to the article, the primary analysis was to use a one-sided t-test of mean changes in emotion dysregulation, with linear mixed-effects models reserved for exploratory analyses. In the submitted manuscript, regression models are presented as primary analysis and mixed-effects models are exploratory analyses. While I agree that regression and - especially - mixed-effects modeling represent a more rigorous and appropriate approach than a one-sided t-test, the discrepancy between the pre-specified protocol and the reported analytic strategy raises some concerns.
The authors should explicitly justify these deviations, and provide either the original protocol submitted to IRB or a transparent account of all changes to the analysis plan:

- We appreciate and agree with the reviewer's observation and welcome the opportunity to clarify this point. As a team, we carefully considered whether to retain the analytic approach specified in the preregistered protocol or to adopt a more appropriate framework once an intervention scientist with evaluation expertise joined the team. We ultimately prioritised methodological rigour by implementing an ANCOVA framework, which is better aligned with contemporary recommendations for analysing randomised trial data. Linear mixed models were also prespecified as exploratory analyses in the BMJ Open protocol. If the reviewers feel it would be helpful, we can provide the original preregistered t-test analyses in the supplementary materials; however, because these analyses are highly overlapping with the ANCOVA models reported here, we have not included them by default.
- We also recognize that deviating from the preregistered analytic plan is not ideal. To ensure transparency, we included in the original submission a supplementary table detailing all deviations from the preregistered protocol's analysis plan and justifications for each change; this table is explicitly referenced in the *Methods* section. After reviewing formatting guides explaining a preference for Tables not to be referenced from the Methods section, we have updated this table to be presented in-text. Please see the new **Deviations from pre-registered analysis plan** section (pages 45-47).

2. The manuscript does not clearly describe whether main effects were included alongside the reported interaction terms. The provided Tables are quite difficult to read, with respect to typical reporting uses. An interaction term is only interpretable relative to the inclusion of the corresponding main effects; otherwise, the model implicitly assumes that the predictors have no effect when the other variable is set to zero.
Clarification is needed on whether the regression and linear mixed-effects models included the appropriate main effects and how these were coded.

- We thank Reviewer 2 for this valuable clarification. We agree that the tables originally submitted were difficult to interpret and have therefore revised them substantially. To improve clarity, we have expanded Tables X and Y so that each model is presented in full. For every outcome, the tables now report the unstandardized coefficients (β), standard errors, 95% confidence intervals, *t*-values, *p*-values, and partial η^2 with corresponding confidence intervals for all predictors and interaction terms. These revisions make it explicit that all relevant main and interaction effects were modeled and reported.
- We also confirm that all moderation models included the main effects of both variables (condition and gender identity) in addition to their interaction term. The **Methods** section and table notes have been updated to specify that *condition* was coded 0 = Waitlist Control and 1 = Purrble Treatment, and *gender identity* was coded 0 = Cisgender and 1 = TGD. This ensures that each β term is interpretable and that the models are clearly aligned with the reviewer's feedback.
- We also confirm that all mixed-effects models included the corresponding main effects (Week and condition) and their interaction term (Week × condition), along with covariates for gender identity and age. These model specifications are explicitly detailed in the Supplementary Materials table reporting the LME model results.

3. Line 133 and Table 2: The reporting is ambiguous. If p-values are presented, these are inferential results and not purely descriptive statistics.
It should be stated clearly what hypothesis is being tested and what the reported p-values represent.

- We thank Reviewer 2 for this helpful clarification and for referencing the specific lines, which made the comment simple and straightforward to address both here and throughout the review.
  The original wording referenced was indeed confusing and implied that inferential results were being presented as descriptive statistics. Our intent was simply to note our general approach to reporting statistical results (i.e., that all significant effects ($p < .05$) are reported and interpreted, whereas marginal effects ($p < .10$) are reported without interpretation). To avoid confusion, we have moved this statement to the *Data Analysis* subsection of the Methods, where it more appropriately provides context for our reporting conventions rather than appearing to describe the contents of the descriptive Table.

4. *Lines 148–149 ("approached significance"):*
This phrase is misleading and should be avoided. Results that are not statistically significant should be reported as such. For instance, the difference in baseline loneliness between completers and those lost to follow-up (d = –0.75, 95% CI [–1.60, 0.09], p = .079) can be described as relatively large in magnitude but not statistically significant

- We thank the reviewer for noting this. This phrasing was an artifact from an earlier draft and should not have appeared in the submitted version, as the loneliness analyses were removed during revisions. We have deleted this sentence entirely and apologize for the oversight.

5. 163–165. The model under discussion can be written as:
$$Y = \beta_0 + \beta_1 ,\text{Condition} + \beta_2 ,\text{Gender} + \beta_3 (\text{Condition}\times\text{Gender}) + \varepsilon,$$
where Condition = 0 (waitlist), 1 (Purrble), and Gender = 0 (cisgender), 1 (TGD).
Under this coding: β_1 = the treatment effect for cisgender participants (since Gender = 0), β_2 = the difference between TGD and cisgender participants in the waitlist group (main effect of gender), β_3 = the difference in treatment effect for TGD compared to cisgender.
Thus, the treatment effect for cisgender youth is given by β_1, while for TGD youth it is $\beta_1 + \beta_3$.
The statement that "condition was not a significant predictor for TGD participants" correctly refers to this combined effect, but it does not address the gender main effect β_2. Moreover, the text risks suggesting that cisgender participants had no post-test difficulties, when the correct interpretation is that they had fewer difficulties relative to controls, conditional on baseline. I recommend clarifying that cisgender participants improved compared to waitlist, while the effect was not significant among TGD participants, and that baseline gender differences are captured separately by β_2.
As mentioned before, the tables should probably be revised to provide a non-ambiguous result.

- We thank the reviewer for this detailed and constructive comment. The specific breakdown of the model coefficients (β_1, β_2, β_3) was extremely helpful, and we have substantially revised Section 2.4.1 to address these points directly. Specifically:
    - To address the point that we had not reported the main effect of gender (β_2, the difference between TGD and cisgender participants in the waitlist group), we have now explicitly stated in the revised text (Section 2.4.1, paragraph 3): "There was no main effect of gender identity." The table also has been expanded so that this effect can be examined directly.
    - To correct the potential misinterpretation that cisgender participants had no post-test difficulties (rather than *fewer* difficulties relative to controls), the revised text now reports the full simple slopes analysis:
        - We clarify the significant conditional effect for cisgender participants (β_1): "Simple slopes showed a significant benefit of Purrble among cisgender participants ($\beta = -5.01$, SE = 1.33, $p < .001$)."

        - We clarify the non-significant conditional effect for TGD participants ($\beta_1 + \beta_3$): "In contrast, the conditional treatment effect for TGD participants was not significant (β = −1.08, SE = 1.32, t = −0.82, p = .42)."
- To further remove ambiguity and show this is a *relative* improvement, we also added the estimated marginal means to the text (e.g., "cisgender: Purrble M = 23.6 vs. waitlist M = 28.6..."), in addition to a table with marginal means, which clearly illustrates the comparison.
- Finally, as suggested, we have heavily revised Table 3 and Table 4 in the main text to present these interaction and Table 3 in Extended Data to present simple slope results in a clear and non-ambiguous format, supplementing the narrative in the main text. Notably, these changes were also applied to subsequent sections.

6. l. 179–181. The same interpretative logic applies. The significant reduction in anxiety among cisgender youth reflects β_1, while the non-significant effect among TGD participants reflects $\beta_1 + \beta_3$. This should not be conflated with the main effect of gender (β_2).
Please revise the wording accordingly.

- Please see our response above (#5).

7. l. 195–198. Again, the condition effect for cisgender is given by β_1, and for TGD by $\beta_1 + \beta_3$.
If the interaction was not significant for depression, this should be stated explicitly, and the interpretation revised to avoid conflating the non-significant condition effect in TGD participants with the absence of gender differences overall.

- We have explicitly clarified that the interactive effect was not significant for depression.
- Please see our response above (#5).
- **Associated Text:**
    - **Results (page 13):** To test whether the effect on depressive symptom severity was moderated by gender identity, we fit a second ANCOVA that included a Condition × Gender Identity interaction term (see Table 4). The interaction was not significant (β = 2.54, SE = 1.42, p = .076; partial η^2 = .023).
    - **Discussion (page 16-17):** No moderation was observed for depression.

8. The manuscript acknowledges that the use of a waitlist control is a limitation, and I agree this is an important point. However, it is not clear why a waitlist design was chosen instead of alternatives such as treatment-as-usual, an active comparator, or a placebo control (e.g., a neutral interactive toy):

- Thank you for this comment. We have clarified the rationale for the waitlist design and how it relates to TAU and potential active/placebo comparators. As described in the revised Public and Patient Involvement section, the trial was co-designed with LGBTQ+ youth advisors (Sprouting Minds), who strongly favoured a waitlist control as the most ecologically valid and ethically acceptable option, reflecting real-world experiences of waiting for mental health services while ensuring eventual access to Purrble for all participants. We also recognised that our original framing did not adequately describe the active component common to both arms; we have therefore reframed the comparison as "Purrble plus safety planning" versus "safety planning only," and explicitly describe safety planning as consistent with treatment-as-usual. At the same time, we now more clearly acknowledge in the Limitations that the "waiting for Purrble" element can introduce expectancy and engagement differences, and we emphasize that findings are provisional until replicated with more robust comparators (e.g., active control or a feasible placebo device).
- Please see our first response to Reviewer 1 for a full explanation as well as associated text.

9. Related to the point above, since the intervention is tangible and novel (a robot that responds interactively), it may have nonspecific psychological effects (attention, novelty, comfort) that are not controlled for. A neutral interactive toy or "sham" device would help disentangle these effects.

- Thank you for this comment, we have included this as a limitation within the manuscript and also expanded the discussion related to the difficulties of identifying the "active components" of Purrble in order to develop a sham device.
- **Associated Text (page 18):** . Third, there was no sham or placebo device to isolate Purrble-specific mechanisms (e.g., socially assistive feedback) from non-specific factors such as novelty or having any comforting object… Future trials would benefit from more active control conditions, such as clearly documented treatment-as-usual comparators[47] or, where feasible, attention-matched or placebo device controls that more stringently test the added value of the socially assistive components of Purrble. A key challenge for developing an appropriate control lies in disentangling which specific functionalities (e.g., tactile feedback, responsive social cues, physical comfort, routine use) most contribute to these in-the-moment regulation effects, and whether these mechanisms operate similarly across users. Because these components are interwoven in the device's interaction model, creating a credible placebo or matched alternative that preserves the nonspecific elements while removing the active mechanisms remains methodologically complex. Further, a larger, sufficiently powered trial is required to establish whether the use/deployment of Purrble could be effective in preventing the escalation of emotional distress into self-harm thoughts and behaviours, a potential effect derived from our pilot work (20).

10. The intervention requires active use of Purrble, but engagement levels vary. Participants who are more motivated or have stronger support systems may use the device more and also have better outcomes, independent of the device itself. Without a measure of adherence or engagement in the main analyses, it is hard to separate the effect of Purrble from the effect of engagement.

- This is a very interesting point, and one we will try to carefully address, as the novel nature of Purrble made conceptualizing engagement complex.
  Specifically, unlike session-based therapies where exposure (attending all sessions) is the mechanism for learning, Purrble is need-contingent and just-in-time: the relevant question is whether youth used it *when* they needed it, as opposed to how much they used it at all. Consequently, frequency/dose may not index beneficial exposure, and a decline in routine use can even be compatible with benefit (e.g., fewer distress episodes requiring use). For these reasons, we did not include engagement in our main effects models.
  To address this and another comment, however, we conducted a post-hoc analysis in which we assessed the perceived engagement/fit with Purrble weekly using with the The Twente Engagement with Ehealth Technologies Scale (TWEETS), which captures behavioral, cognitive, and affective fit rather than dose. TWEETS declined over time and declined more steeply for TGD youth, which we report descriptively to contextualize subgroup patterns.
- **Associated Text**
  - **Results (page 15-16):** To elucidate potential reasons for the differential efficacy observed between sexual and gender minority youth, we examined whether perceptions of Purrble engagement/fit differed by gender identity over time. While there was no baseline difference in perceived engagement/fit of Purrble by gender identity ($b = -0.06$, $SE = 0.10$, $p = .54$), a significant Week × Gender Identity interaction ($b = 0.017$, $SE = 0.006$, $t(531) = 2.93$, $p = .004$) indicated that Purrble engagement/fit waned significantly faster for TGD youth. Specifically, TGD youth showed a steeper decline in perceived engagement/fit (simple slope $b = -0.056$, 95% CI [−0.073, −0.040]) compared with their cisgender peers ($b = -0.022$, 95% CI [−0.038, −0.005]).
  - **Discussion (page 17):** In post-hoc analyses, TGD and cisgender participants did not differ at baseline in perceived Purrble fit. However, over the intervention period, TGD youth showed a steeper decline in perceived fit than cisgender youth. Future qualitative and mixed-methods work could clarify how TGD youth interpret and use Purrble in daily life, and which design features may need adaptation.

Reviewer #3 (Remarks to the Author):

A.This study reports on findings from an RCT of the Purrble intervention, designed to improve emotion regulation and transdiagnostic risk. The findings indicate support for Purrble in cisgender sexuality diverse youth in terms of improved emotion regulation and reduce depression and anxiety.
E. While the conclusions are appropriate, I would caution the authors from stating that Purrble is effective for LGBTQA+ young people if there is limited evidence to support its efficacy with trans and gender diverse youth - perhaps it would be more appropriate to use the term cisgender sexuality diverse young people?

- Thank you very much for this crucial suggesion. We agree and have revised efficacy statements to refer specifically to cisgender sexual-minority youth, with transgender and gender-diverse (TGD) results described as exploratory and in need of more research. We now avoid umbrella claims about “LGBTQ+ youth” when discussing effects. “LGBTQ+” is retained only to describe the enrolled sample. We also added language noting the trial was not powered for subgroup efficacy in TGD youth and that dedicated, co-designed trials are needed.

B. This study represents a novel and innovative contribution to the literature and a potential scalable intervention to support emotional regulation in young people. While there are emerging digital and AI interventions for this population, to my knowledge there are no other socially assistive robots being evaluated or freely available for this population.

- Thank you for your comment; in keeping with other reviewers’ comments, we have more closely incorporated broader digital intervention literature concerning LGBTQ+ young people. Please see our response to reviewer 1 under “References” for a full response to this query.

C. The study utilises rigorous methodology and reporting, with strong attention to CONSORT guidelines and justification for deviations. My main concern is around the validity of measures for LGBTQA+ young people, the role that LGBTQA+ youth played in the context of Sprouting Minds, whether the ASIST training was adapted for LGBTQA+ youth and if any adaptations were made for the safety plans. In short, while the intervention was applied to LGBTQA+ young people, I can't see much evidence of tailoring the study to the population, or a rationale as to why this wasn't needed.

- This study was supported by members of *Sprouting Minds*, an advisory group of young people with lived experience of mental health difficulties and self-harm, including one member who identifies as LGBTQ+. To clarify this, the introduction to Public and Patient Involvement has been revised to discuss the nature of their involvement.

- Study decisions were reviewed collaboratively with these advisors, with particular emphasis on co-designing safeguarding protocols. Safeguarding measures were tailored specifically for LGBTQ+ participants, including:
    - using LGBTQ+-affirmative prompts during safety briefings and any reactive safeguarding (e.g., “Is there someone you feel comfortable sharing your difficulties with?”) and signposting to LGBTQ+ helplines (e.g., Switchboard);
    - including signposting to LGBTQ+ organisations in all surveys, alongside GP contact recommendations; and
    - considering previous literature (Williams et al., 2021), and following consultation with LGBTQ+ advisors and researchers, and the ethics committee, the named contact for reactive safeguarding was not required to be a parent or guardian, to protect participants who may lack a supportive home environment.
- However, we did not take specific precautions around measurement validation, as there was insufficient psychometric work conducted at the time of the trial to be able to adequately do so.
- **Associated Text (page 35):** “This project is supported by members of Sprouting Minds—an advisory group comprising young individuals with lived experience of poor mental health, specifically involved in Digital Youth research initiatives. A subgroup of advisors from the wider Sprouting Minds network chose to engage more closely with this project due to shared identity characteristics and personal relevance to the study population. Building on their foundational involvement in prior related research (20), their input substantially shaped key design decisions throughout this trial design.”

D. To my knowledge, analyses were robust and appropriate, with justification clearly outlined.

F. I understand that additional information is provided in the supplementary materials, however, I would like to see a more detailed explanation of the reasoning for why trans and gender diverse young people were not responsive to Purrble. It also makes sense to suggest this as an area for further exploration in future research.

- Due to some ambiguity surrounding the interpretation of the process analysis presented in the supplementary materials and lack of details included in the main text, we have chosen to withdraw the qualitative process analysis and any conclusions derived from it. This decision will allow us to present the process analysis findings in greater depth within a separate publication and ensure that our conclusions within this manuscript are clearly grounded in published evidence.
  To address this comment, however, we conducted a post-hoc analysis in which we assessed the perceived engagement/fit with Purrble weekly using with the The Twente Engagement with Ehealth Technologies Scale (TWEETS), which captures behavioral,

cognitive, and affective fit rather than dose. TWEETS declined over time and declined more steeply for TGD youth, which we report descriptively to contextualize subgroup patterns. We discuss the need for future mixed-methods and qualitative research to interpret these differences.

- **Associated Text**
  - **Results (page 15-16):** To elucidate potential reasons for the differential efficacy observed between sexual and gender minority youth, we examined whether perceptions of Purrble engagement/fit differed by gender identity over time. While there was no baseline difference in perceived engagement/fit of Purrble by gender identity ($b = -0.06$, $SE = 0.10$, $p = .54$), a significant Week × Gender Identity interaction ($b = 0.017$, $SE = 0.006$, $t(531) = 2.93$, $p = .004$) indicated that Purrble engagement/fit waned significantly faster for TGD youth. Specifically, TGD youth showed a steeper decline in perceived engagement/fit (simple slope $b = -0.056$, 95% CI [−0.073, −0.040]) compared with their cisgender peers ($b = -0.022$, 95% CI [−0.038, −0.005]).
  - **Discussion (page 17):** In post-hoc analyses, TGD and cisgender participants did not differ at baseline in perceived Purrble fit. However, over the intervention period, TGD youth showed a steeper decline in perceived fit than cisgender youth. Future qualitative and mixed-methods work could clarify how TGD youth interpret and use Purrble in daily life, and which design features may need adaptation.

Further, what was the rationale for using a Cohen's d of 0.4 in your power analysis?

- As this was a preliminary trial for Purrble our power analysis was determined by previous Purrble studies and digital intervention literature, this was spread between LGBTQ+ targeted interventions and young people more broadly. Given that this was not a definitive trial, we were uncertain about the effect sizes that should be expected given the novelty of the intervention and therefore made a calculated guess.
- **Associated Text (page 44):** "A medium effect size of 0.4 was selected as a preliminary exploration based on previous intervention research with and without Purrble given the uncertainty of the novel intervention within this population."

G. Appropriate referencing

H. The manuscript was very well written with a clear rationale for the study and justification for methodological and analytic decisions. The abstract was clear and accurate (though reference to

Purrble supporting LGBTQA+ youth in general may need to be toned down to reflect benefits to the cisgender cohort specifically).

- Thank you for your kind words. We have clarified in our abstract that this might be an efficacious intervention for cisgender participants.
- **Associated Text (Abstract):** In exploratory analyses, this benefit was evident for cisgender participants but not for transgender or gender diverse participants... Purrble may offer a novel, scalable intervention to complement existing therapeutic approaches to support cisgender LGBTQ+ youth in their emotion regulation.

Response to Editorial and Reviewer Comments

We thank the Editors and Reviewer #3 for their careful evaluation of our manuscript and for the constructive guidance provided. We have revised the manuscript extensively in response to all comments. Below, we provide a detailed, point-by-point account of the revisions made. All changes are reflected in the tracked version of the manuscript.

Please define all abbreviations at first use and minimise any unnecessary abbreviations in the manuscript.

- All abbreviations have now been reviewed and defined at first use.
- Two abbreviations that were judged to be unnecessary for readability have been removed.
- Four instances where abbreviations appeared before definition have been corrected.
- We further reduced abbreviation density in narrative sections to improve clarity.

Please remove Figure placeholders such as "insert Figure 1".

- All figure placeholders have been removed from the manuscript.

In the data availability statement, please provide more details regarding how the dataset can be made available, the procedures to apply, and the timelines for a decision to grant access upon request by a researcher.

- The Data Availability Statement has been substantially revised.
- We now specify that a minimally deidentified dataset (excluding demographic and other potentially identifying variables) has been deposited in Code Ocean and is publicly available.
- We describe the procedure for requesting access to the full analytic dataset, including contact details, the requirement for a data use agreement, and explicit timelines (data provided within one month of agreement completion).

We do not allow "code available upon request". Please either deposit the code in a repository or provide more information on how the code can be accessed.

- All analysis code has been deposited in Code Ocean, the repository supported by *Nature Medicine*.
- The Code Availability Statement has been updated accordingly, and all "available upon request" language has been removed.

Line 397–398: please remove "correspondence and requests…" from the competing interests section.

- This language has been removed from the Competing Interests section.

- Relevant correspondence information has been appropriately incorporated into the Data Availability Statement.

Please remove editorialising terms/language throughout the manuscript when describing the study and findings (e.g., “first”, “novel”, “largest/most comprehensive”, “clearly”).

- The term **“first”** has been removed throughout.
- The term **“novel”** has been removed when describing the study and findings.
- Terms such as **“clearly”**, **“largest”**, and **“most comprehensive”** were not used to describe findings and therefore required no changes.
- Language across the manuscript has been reviewed and revised to ensure a descriptive, non-editorial tone grounded in reported results and effect sizes.

Response to Reviewer #3 Comments

Comment C - the revised text describing the role of the advisory group doesn't fully specify the role of LGBTQA+ lived experience within the group. You note that only 1 member of the advisory group identified as LGBTQA+ - this should be explicit (or at least noted as a % of the group) and could be mentioned in the limitations section. In particular, trans membership of an advisory group would be critical to future research in this space.

- Thank you for this important point. We agree that the role of lived experience within the advisory group should be specified more clearly. We have revised the manuscript to explicitly note that one member of the advisory group (representing approximately [33%] of the group) identified as LGBTQA+.
- In addition, we have added this point to the Limitations section, noting that broader LGBTQA+ representation, particularly inclusion of trans individuals with lived experience, would be critical for future research in this space. We now explicitly highlight the need for deeper and more diverse lived-experience involvement in subsequent iterations of this work.

Comment F - the revised text on page 44 outlining the rationale for effect size should include citations to the 'previous intervention research with and without Purrble'.

- Thank you for this suggestion. We have revised the text on page 44 to explicitly reference prior intervention research in this section, including Moltrecht et al. (2021), a meta-analysis of psychological interventions targeting emotion regulation in youth that reported a pooled effect size of $d = 0.36$ (European Child & Adolescent Psychiatry, 30(6), 829–848).

This citation was already included elsewhere in the manuscript; however, we agree that it should also be referenced here to more clearly ground the effect size rationale in prior intervention work. We appreciate the reviewer’s careful reading in noting this and prompting us to improve clarity and consistency.
